# Novel Insights Into the Phylogeny and Biotechnological Potential of *Weissella* Species

**DOI:** 10.3389/fmicb.2022.914036

**Published:** 2022-06-22

**Authors:** Francesca Fanelli, Marco Montemurro, Daniele Chieffi, Gyu-Sung Cho, Charles M. A. P. Franz, Anna Dell'Aquila, Carlo Giuseppe Rizzello, Vincenzina Fusco

**Affiliations:** ^1^National Research Council, Institute of Sciences of Food Production (CNR-ISPA), Bari, Italy; ^2^Department of Soil, Plant and Food Science, University of Bari Aldo Moro, Bari, Italy; ^3^Department of Microbiology and Biotechnology, Max Rubner-Institut, Kiel, Germany; ^4^Department of Environmental Biology, Sapienza University of Rome, Rome, Italy

**Keywords:** *Weissella* spp., phylogenomics, genomics, carbohydrate metabolism, trehalose

## Abstract

In this study, the genomes of the *Weissella* (*W*.) *beninensis, W. diestrammenae, W. fabalis, W. fabaria, W. ghanensis*, and *W. uvarum* type strains were sequenced and analyzed. Moreover, the ability of these strains to metabolize 95 carbohydrates was investigated, and the genetic determinants of such capability were searched within the sequenced genomes. 16S rRNA gene and genome-based-phylogeny of all the *Weissella* species described to date allowed a reassessment of the *Weissella* genus species groups. As a result, six distinct species groups within the genus, namely, *W. beninensis, W. kandleri, W. confusa, W. halotolerans, W. oryzae*, and *W. paramesenteroides* species groups, could be described. Phenotypic analyses provided further knowledge about the ability of the *W. beninensis, W. ghanensis, W. fabaria, W. fabalis, W. uvarum*, and *W. diestrammenae* type strains to metabolize certain carbohydrates and confirmed the interspecific diversity of the analyzed strains. Moreover, in many cases, the carbohydrate metabolism pathway and phylogenomic species group clustering overlapped. The novel insights provided in our study significantly improved the knowledge about the *Weissella* genus and allowed us to identify features that define the role of the analyzed type strains in fermentative processes and their biotechnological potential.

## Introduction

*Weissella* is a genus belonging to the phylum *Firmicutes*, order *Lactobacillales*, and family *Lactobacillaceae*. *Weissella* species are nonspore-forming, catalase-negative, and Gram-positive bacteria with either a coccoid or rod shape. They have a widespread occurrence, being isolated from various ecological niches such as soil (mainly *Weissella soli*) (Chen et al., 2005), sediments of coastal marsh and estuary (Sica et al., 2010), plants (Emerenini et al., 2014), and foods. Their presence in food matrices and many spontaneous fermentation processes of vegetables, dairy, and cereals (Wang et al., 2011; Zannini et al., 2013, 2018; Mun and Chang, 2020) reveals their role in these fermentative processes, in which they exert an influence on the quality, and especially on the texture of foods. In addition, they have been isolated from the gut, oral cavity, breast milk (Martín et al., 2007; Albesharat et al., 2011), and urogenital and gastrointestinal tracts of humans (Lee et al., 2012), as well as from many animals, such as giant pandas and rainbow trout, and from the skin, milk, and gastrointestinal tracts of vertebrates (Xiong et al., 2019; Mortezaei et al., 2020; Wang et al., 2020).

The presence of these species as common inhabitants of the human intestine (Lee et al., 2012) and animal feces (Cai et al., 1998; Beasley et al., 2006; Muñoz-Atienza et al., 2013), as well as their occurrence in a variety of food matrices, has suggested their potential use as probiotics (Teixeira et al., 2021). This potential probiotic activity is supported by observations that some *Weissella* species can overcome the gastric barrier (Wang et al., 2008) and produce antimicrobial compounds such as bacteriocins (Srionnual et al., 2007; Masuda et al., 2012). For example, *W. cibaria* has been reported to inhibit biofilm formation *in vitro* and the proliferation of the main bacterial pathogens in dental caries and upper respiratory infections (Yeu et al., 2021), and has been studied for its potential anti-inflammatory activity against lipopolysaccharide stimulation (Yu et al., 2019), while *W. viridescens* was proposed as a potential probiotic for the skin (Espinoza-Monje et al., 2021). Furthermore, potential probiotic features of this genus include the reduction in depressive-like behavior (Sandes et al., 2020), the influence on gut permeability and intestinal epithelial regeneration (Prado et al., 2020), and the antagonistic activity against common human pathogens (Afolayan et al., 2017; Choi et al., 2018; Yu et al., 2019).

The capacity of this genus to grow at a wide range of temperatures, water activity levels, and pH (Ricciardi et al., 2009) increases its promising biotechnological potential, mainly attributed to the ability to produce exopolysaccharides (EPS) (Fusco et al., 2015). This has already promoted their use as starter cultures in dairy industries and sourdough fermentation (Galle et al., 2010), especially in the preparation of gluten-free baked products (Li et al., 2021). *W. cibaria* (Galli et al., 2020) and *W. confusa* have been largely studied for their ability to produce high amounts of dextrans, which enhance the softness of fresh bread (Wolter et al., 2014), and also for improving the textural properties of gluten-free bread (Montemurro et al., 2021).

The genus *Weissella*, originally described by Collins et al. (1993), to date comprises 21 validated species. The last taxonomic classification of *Weissella* species had been proposed by Fusco et al. (2015) who, updating the phylogenetic analysis performed by De Bruyne et al. (2010), clustered the species in 5 defined branches. These were designated as *W. kandleri* (including *W. kandleri, W. soli, W. diestrammenae, W. koreensis*, and *W. oryzae*), *W. confusa* (comprising *W. cibaria* and *W. confusa*), *W. halotolerans* (*W. halotolerans, W. ceti, W. viridescens, W. minor*, and *W. uvarum*), *W. paramesenteroides* (*W. thailandensis, W. hellenica*, and *W. paramesenteroides*), and *W. beninensis* (*W. beninensis, W. fabalis, W. fabaria*, and *W. ghanensis*) species groups, respectively.

Since 2015, seven additional *Weissella* species have been described, including *W. bombi* (Praet et al., 2015), *W. coleopterorum* (Hyun et al., 2021), *W. cryptocerci* (Heo et al., 2019), *W. muntiaci* (Lin et al., 2020), *W. sagaensis* (Li et al., 2020), and *W. jogaejeotgali* (Lee et al., 2015). According to Kwak et al. (2019), *W. jogaejeotgali* is a later heterotypic synonym of *W. thailandensis*, while Ennahar and Cai (2004) reported that *W. kimchii* (Choi et al., 2002) is a later heterotypic synonym of *W. cibaria* (Björkroth et al., 2002).

In this study, we sequenced the genomes of *W. beninensis, W. diestrammenae, W. fabalis, W. fabaria, W. ghanensis*, and *W. uvarum* type strains. These were the only *Weissella* species whose assemblies were not available at the time this study was performed. Based on our genomic analysis, we updated the taxonomic classification of *Weissella* species. We also phenotypically characterized the newly sequenced strains, evaluating their capability to metabolize different carbohydrates, while identifying the genetic determinants encoding these enzymatic activities.

## Materials and Methods

### Strain Info and Culture Conditions

*Weissella beninensis* LMG 25373^T^, *W. diestrammenae* DSM 27840^T^, *W. fabalis* LMG 26217^T^, *W. fabaria* LMG 24289^T^, and *W. ghanensis* DSM 19935^T^ were purchased from DSMZ (website: http://www.dsmz.de) and BCCM/LMG (http://bccm.belspo.be/db/lmg_search_form.php), whereas the *W. uvarum* B18NM42 type strain (=DSM 28060^T^) was kindly provided by Dr. Aspasia Nisiotou from the Institute of Technology of Agricultural Products, Hellenic Agricultural Organization Lykovrisi, Greece. The strains were grown as described by Fusco et al. (2011).

### Whole-Genome Sequencing

A single colony of each strain was inoculated in 10 ml of MRS (de Man, Rogosa, and Sharpe) broth (Oxoid, Italy) and incubated overnight at 30°C. Two mL of each broth culture were washed in Tris-HCl (10 mM, pH 7.5) and resuspended in 500 μl of the same buffer. Genomic DNA was extracted using the peqGOLD bacterial DNA kit (Peqlab, Erlangen, Germany) according to the manufacturer's instructions. The integrity, purity, and quantity of DNA were assessed using agarose gel electrophoresis, Nanodrop photometer (Peqlab), and Qubit 3.0 fluorometer (Life Technologies). To prepare sequencing libraries, the Illumina TruSeq Nano DNA LT Library Prep Kit (MiSeq v3-kit) (Illumina, San Diego, USA) was used according to the manufacturer's instructions, and then sequenced on the Illumina MiSeq platform using the 2 × 250 pair procedure. Reads were then trimmed with the NxTrim (V2) (O'Connell et al., 2015) and the Trimmomatic (Bolger et al., 2014), and then *de novo* assembly was performed using SPAdes version 3.10.1 (Bankevich et al., 2012). The whole-genome shotgun projects were deposited at DDBJ/ENA/GenBank under the accessions JAGMVS000000000 for *W. beninensis* LMG 25373^T^, JAGMVT000000000 for *W. diestrammenae* DSM 27840^T^, JAGMVU000000000 for *W. fabalis* LMG 26217^T^, JAGMVV000000000 for *W. fabaria* LMG 24289^T^, JAGMVW000000000 for *W. ghanensis* DSM 19935^T^, and JAGMVX000000000 for *W. uvarum* B18NM42^T^. The versions described in this study are JAGMVS010000000 for *W. beninensis* LMG 25373^T^, JAGMVT010000000 for *W. diestrammenae* DSM 27840^T^, JAGMVU010000000 for *W. fabalis* LMG 26217^T^, JAGMVV010000000 for *W. fabaria* LMG 24289^T^, JAGMVW010000000 for *W. ghanensis* DSM 19935^T^, and JAGMVX010000000 for *W. uvarum* B18NM42^T^.

### Bioinformatic Methods

The completeness of the assemblies was assessed by identifying a set of essential genes, which are typically present in a single copy in almost all prokaryotic genomes, by using MiGA (Rodriguez-R et al., 2018) and estimating the percentage of their presence in the genome. The quality scores were then calculated as the completeness percentage minus five times the contamination percentage.

Proteins were predicted by using the Prokaryotic Genome Annotation Pipeline (Tatusova et al., 2016) and PROKKA pipeline (Seemann, 2014) implemented in the Galaxy platform (Galaxy Version 1.14.6 + galaxy0; Afgan et al., 2018). Functional classification, subsystem prediction, and metabolic reconstruction comparison were performed using the RAST server (Aziz et al., 2008). All the protein sequences used in this study were retrieved from GenBank (NCBI). The homology-based relationship of *Weissella* strains toward selected proteins was determined using the BLASTP algorithm on the NCBI site (http://blast.ncbi.nlm.nih.gov/Blast.cgi). Gene models were manually determined, and clustering and orientation were subsequently deduced for the closely linked genes. Genes were also retrieved by keyword search within the UniProtID entry list obtained by functional annotation and then manually curated.

#### Phylogenetic Analysis

A comparative genomic analysis was performed using strains and genomic assemblies listed in Supplementary Table S1. The 16S rRNA gene sequences were extracted from each genome by a BLASTn search and compared to the type strain sequences retrieved from available assemblies, whose accession numbers are indicated in Supplementary Table S1. An alignment was generated by MUSCLE 3.8.31 run mode using the phylogeny.fr platform (http://www.phylogeny.fr/index.cgi), configured as “A la Carte” Mode (Dereeper et al., 2008). The phylogenetic reconstruction was performed using the neighbor-joining method; the phylogenetic robustness was inferred by a bootstrapping procedure with 500 replications to obtain the confidence value for the aligned sequence dataset. The tree was graphically generated by iTOL version 5.5 (Letunic and Bork, 2019).

The genetic divergence was calculated using the ANI/AAI calculator (Goris et al., 2007; Rodriguez-R and Konstantinidis, 2016), which estimates the average nucleotide/amino acid identity (ANI/AAI) between genomic datasets using both best hits (one-way ANI) and reciprocal best hits (two-way ANI).

A genome-based phylogeny was reconstructed using the Phylogenetic Tree Building Service implemented in the Patric platform (www.patricbrc.org) with *Bifidobacterium bifidum* ATCC 29521^T^ as an outgroup according to Fusco et al. (2015) and the maximum likelihood method RAxML with progressive refinement (Stamatakis, 2014). For each strain, the genomic sequences deposited in GenBank were used for the analysis (Supplementary Table S1).

#### Comparative Genomic Analysis

A comparative analysis of carbohydrate-active enzymes (CAZy) was performed by executing a CAZyme annotation using the dbCAN meta server (http://bcb.unl.edu/dbCAN2/blast.php). The database was searched for preserved domains of all CAZy families, following the protocol proposed by dbCAN (Yin et al., 2012). A heatmap was manually constructed and visualized using the heatmapper web server (www.heatmapper.ca; Babicki et al., 2016), with average linkage as the clustering method and the Euclidean distance measurement method.

A comparative analysis of carbohydrate metabolism was performed by using the pathway reconstruction, annotating proteins with the Kyoto Encyclopedia of Genes and Genome (KEGG) Mapper (Kanehisa and Sato, 2020). A heatmap was again manually constructed based on the protein count in each pathway retrieved for individual genomes, and visualized by using the heatmapper web server (www.heatmapper.ca; Babicki et al., 2016) with average linkage as the clustering method and the Euclidean distance measurement method.

A comparative analysis of SEED subsystems was performed by uploading individual genome sequences to the SEED Viewer Server (Overbeek et al., 2014). Functional roles of RAST annotated genes were assigned and grouped in subsystem feature categories.

### Phenotypic Characterization of *Weissella* Strains

#### Phenotypic Characterization

Phenotypic characterization was carried out as previously described by Fanelli et al. (2020), with some modifications. Biolog AN (Biolog, Inc., Hayward, CA, USA) plates were used to evaluate the consumption of 95 different carbon sources. Briefly, strains were grown in MRS broth (Oxoid, Italy) for 24 h. Cells were then harvested by centrifugation (10,000 rpm for 10 min) and washed two times with sterile phosphate buffer (50 mmol/l pH 7.0). Thereafter, cells were resuspended in sterile physiological saline (0.9 w/v NaCl). Each well of the plates was inoculated with 100 μl of bacterial suspension, adjusted to 65% transmittance. Plates were incubated in an anaerobic jar at 35°C for 24 h as recommended by the manufacturer. Positive reactions were automatically recorded using the MicroStation microplate reader (Biolog) with 590 nm and 750 nm wavelength filters.

#### Statistical Analysis

All analyses were carried out in triplicates. Metabolic fingerprints of *Weissella* strains as determined by the Biolog system were subjected to permutation analyses using the PermutMatrix as described by Fanelli et al. (2020).

## Results

### *In silico* Analysis of *Weissella* Genomes

#### General Features of *Weissella* Genomes

The genomes of the six *Weissella* type strains were sequenced and assembled using the SPAdes software (version 3.10.1), obtaining a total of 76, 33, 39, 40, 106, and 26 contigs (>500 bp) for *W. beninensis* LMG 25373^T^, *W. diestrammenae* DSM 27840^T^, *W. fabalis* LMG 26217^T^, *W. fabaria* LMG 24289^T^, *W. ghanensis* DSM 19935^T^, and *W. uvarum* B18NM42^T^, respectively. The mol% GC content ranged from 35.45% (*W. beninensis* LMG 25373^T^) to 39.53% (*W. ghanensis* DSM 19935^T^), while the assembly length varied from 1,639 Mbp (*W. diestrammenae* DSM 27840^T^) to 2,014 Mbp (*W. ghanensis* DSM 19935^T^) (Table 1). The quality of the assemblies was excellent for all genomes, except *W. fabaria* LMG 24289^T^, for which the assembly quality score was 76.5 (high). The contig N50 values were 57, 163, 166, 143, 54, and 246 kbp for *W. beninensis* LMG 25373^T^, *W. diestrammenae* DSM 27840^T^, *W. fabaria* LMG 24289^T^, *W. fabalis* LMG 26217^T^, *W. ghanensis* DSM 19935^T^, and *W. uvarum* B18NM42^T^, respectively.

**Table 1:** Genomic features of *Weissella* spp. strains.

**Features**	***W. beninensis* LMG 25373^**T**^**	***W. diestrammenae* DSM 27940^**T**^**	***W. fabalis* LMG 26217^**T**^**	***W. fabaria* LMG 24289^**T**^**	***W. ghanensis* DSM 19935^**T**^**	***W. uvarum* B18NM42^**T**^**
Genome size (bp)	1,831,593	1,639,510	1,958,377	1,898,123	2,013,964	1,682,559
GC (%)	35.45	39.22	36.40	38.14	39.53	39.84
Number of contigs	76	33	39	40	106	26
Completeness % (essential genes found)	100% (106/106)	99.1% (105/106)	100% (106/106)	100% (106/106)	100% (106/106)	99.1% (105/106)
Quality	90.5 (excellent)	89.6 (excellent)	81.0 (excellent)	76.5 (high)	81.0 (excellent)	80.01 (excellent)
Contig N50 (bp)	57,467	162,981	143,119	166,541	53,895	246,285
Genes (total)	1,867	1,662	1,962	1,920	2,043	1,731
Genes (coding)	1,745	1,551	1,877	1,840	1,968	1,659
Coding density	85.40	87.07	86.28	87.31	87.15	87.59
RNA	61	57	58	66	55	60
rRNAs	1, 1, 2 (5S, 16S, 23S)	1, 1 (16S, 23S)	2, 2, 2 (5S, 16S, 23S)	2, 1, 2 (5S, 16S, 23S)	1, 1, 1 (5S, 16S, 23S)	1, 2, 1 (5S, 16S, 23S)
tRNAs	53	52	49	58	49	53
ncRNAs	4	3	3	3	3	3
Pseudo genes (total)	61	14	27	14	20	12

#### Phylogenetic Analysis

**Figure 1:**
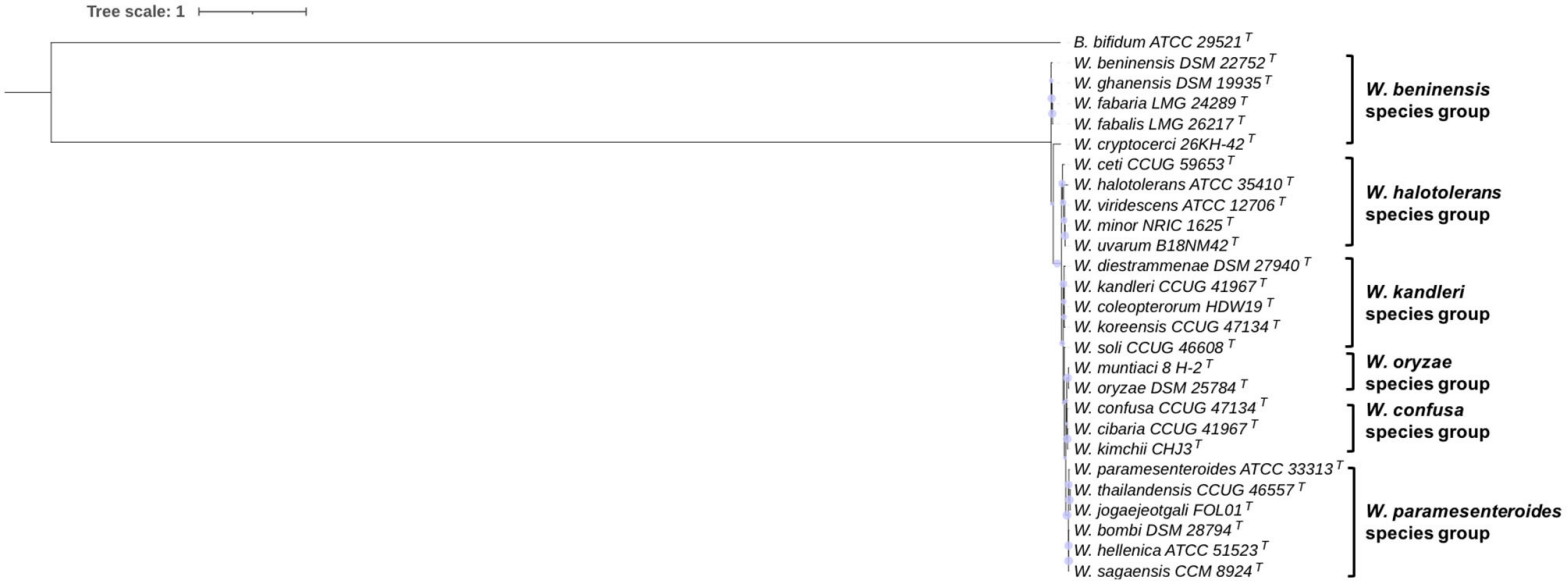
16S rRNA gene-based phylogeny of *Weissella* species. Phylogeny was performed using the neighbor-joining method; phylogenetic robustness was inferred by a bootstrapping procedure from 500 replications to obtain the confidence value for the aligned sequence dataset. *Bifidobacterium bifidum* ATCC 29521^T^ was used as an outgroup. The tree was drawn to scale, with branch lengths measured in the number of substitutions per site. Scaled circles are representative of bootstrap values.

The 16S rRNA gene sequence-based phylogeny is shown in Figure 1. According to the reconstructed tree, *Weissella* species can be clustered into six different species groups. The first group is constituted by *W. thailandensis, W. bombi, W. paramesenteroides, W. hellenica*, and *W. sagaensis*; a second group is formed by *W. cibaria* (*W. kimchii*) and *W. confusa*, which have a 16S rRNA gene sequence identity of 99.3%. The same percentage is shared by *W. oryzae* and *W. muntiaci*, which can be placed in a third species group. *W. soli* occurs close to these couples in the phylogenetic tree and has the highest 16S rRNA gene sequence identity with *W. muntiaci* (97.35%). *W. ceti, W. halotolerans, W. minor, W. uvarum*, and *W. viridescens* are clustered in a fourth species group with an average 16S rRNA gene sequence identity of 95.1%. *W. diestrammenae, W. kandleri*, W. *coleoptorum*, and *W. koreensis* clustered in the fifth group with a shared average 16S rRNA gene sequence identity of 96.8%. *W. cryptocerci, W. beninensis, W. ghanensis, W. fabalis*, and *W. fabaria* are located outside this large clade and share an average sequence identity of 96%, with *W. fabaria* and *W. fabalis* clustering at a value of 99.41%.

The ANI analysis results are reported in Supplementary Table S2. The species sharing the highest ANI values are *W. hellenica* and *W. sagaensis* (89.73%), followed by *W. ghanensis* and *W. fabalis* (85.63%), *W. beninensis* and *W*. k*andleri* (68.08%), while *W. halotolerans* with either *W. beninensis* (67.29%) or *W. ghanensis* (67.01%) are the most divergent species within the genus.

The matrix of AAI values is shown in Supplementary Table S3. Confirming the ANI analysis results, the most closely related species sharing the highest AAI values were *W. hellenica* and *W. sagaensis* (92.75%), followed by *W. hellenica* and *W. bombi* (89.45%). The lowest AAI value was shared between *W. muntiaci* and *W. cryptocerci* (51.91%).

**Figure 2:**
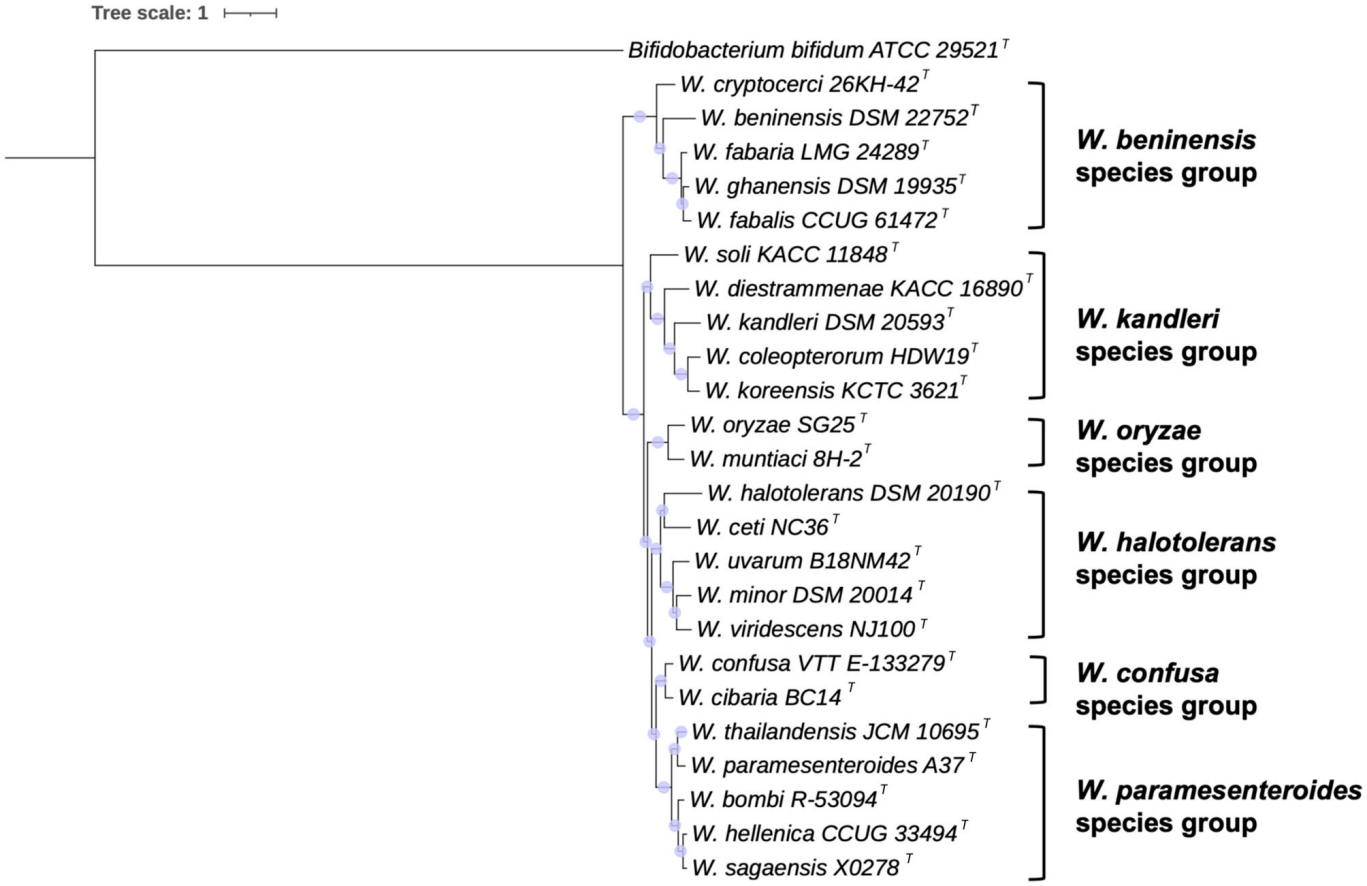
Genome-based phylogeny of *Weissella* species. The tree was inferred by using the maximum likelihood method RAxML with progressive refinements. *Bifidobacterium bifidum* ATCC 29521^T^ was used as an outgroup. The tree is drawn to scale. Support values are represented by scaled circles at each node.

A genome-based phylogeny was inferred from RAxML analysis. The results are shown in Figure 2. Partially confirming the clustering obtained by 16S rRNA gene sequence phylogeny, six species groups could be identified and named as *W. beninensis, W. kandleri, W. oryzae, W. confusa, W. paramesenteroides*, and *W. halotolerans* species groups. The *W. beninensis* group includes *W. beninensis, W. cryptocerci, W. fabalis, W. fabaria*, and *W. ghanensis*. The *W. kandleri* group comprises *W. kandleri, W. diestrammenae, W. koreensis, W. soli*, and *W. coleoptorum*. The *W. oryzae* group includes *W. oryzae* and *W. muntiaci*. The fourth species group, named the *W. confusa* species group, comprises *W. confusa* and *W. cibaria*. The fifth species group, designated as *W. paramestenteroides* by Fusco et al. (2015), includes *W. paramesenteroides, W. hellenica*, and *W. thailandensis*, with the addition of the recently described *W. sagaenisis* (Li et al., 2020) and *W. bombi* species (Praet et al., 2015). The sixth species group, already designated as *W. halotolerans* by Fusco et al. (2015), includes *W. halotolerans, W. ceti, W. uvarum, W. minor*, and *W. viridescens*.

### Genomic and Phenotypic Characterization

#### Carbohydrate Metabolism Comparative Analysis

**Figure 3:**
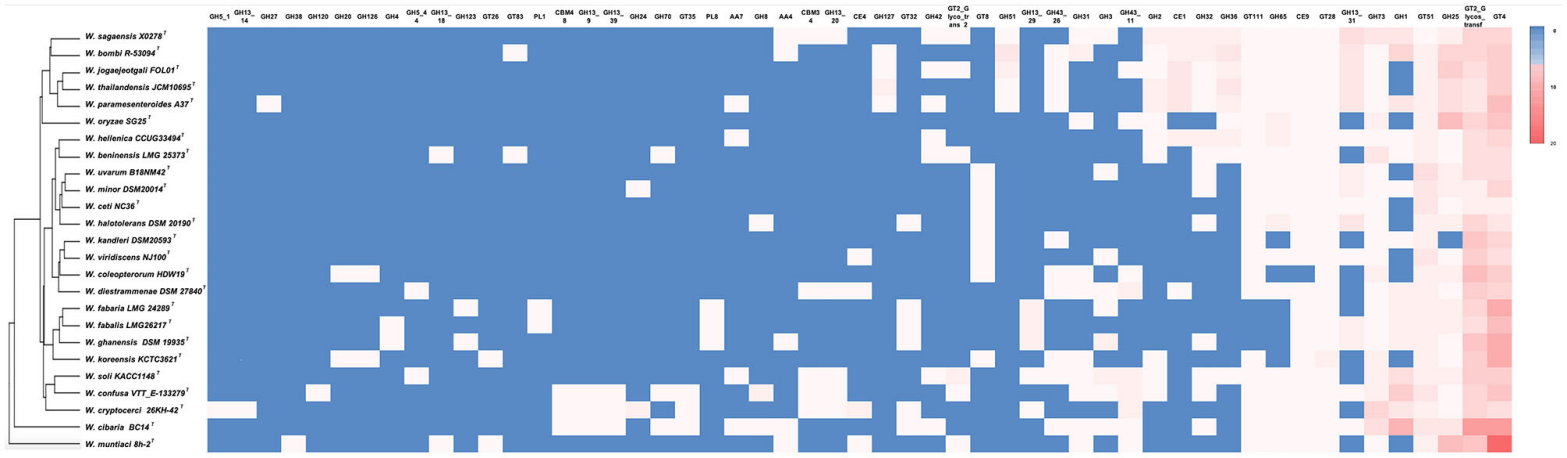
Comparative analysis of CAZymes in *Weissella* species. The heatmap was manually constructed based on CAZymes count in each species and visualized with the average linkage as a clustering method and the Euclidean distance measurement method, providing the resulting dendrogram.

A comparative analysis of CAZy families present in the genomes of *Weissella* spp. is shown in Figure 3. The largest families are represented by the glycosyl transferases (GTs), which catalyze the transfer of sugar moieties from activated donor molecules to specific acceptor molecules, forming glycosidic bonds, followed by the glycosyl hydrolases (GHs). Polysaccharide lyases (PLs) and carbohydrate esterase (CE) are poorly represented, with CE9 and CE1 being the most frequently occurring. The highest number of CAZymes, with 61 annotated proteins, was identified in the genome of the *W. cibaria* type strain, while the lowest number was found in the *W. ceti* type strain, for which only 13 CAZymes could be identified. The *W. bombi* and *W. cibaria* type strains have the highest numbers of GHs (28), while the *W. muntiaci* and *W. cibaria* type strains have the highest number of GTs (29). Figure 3 also shows the clustering of *Weissella* species obtained by analyzing the occurrence and distribution of CAZymes among species.

A detailed analysis of the enzymes involved in the metabolism of carbohydrates of the six strains sequenced in this study is reported in Table 2 and Supplementary Table S4. Regarding the hydrolytic enzymes, 13 GH proteins were annotated in the *W. ghanensis* type strain, while 12 were found in the *W. beninensis* type strain and *W. diestrammenae* type strain each. One FAD-binding protein with an auxiliary activity family 4 domain that catalyzes the conversion of a wide range of phenolic compounds was identified in the *W. ghanensis* type strain. Polysaccharide lyase enzymes were retrieved in the genomic sequences of the *W. ghanensis, W. fabaria*, and *W. fabalis* type strains. One carbohydrate-binding module CBM34 zyme was retrieved in the genomic sequence of the alpha-glycosidase (GH13_20) of the *W. diestrammenae* type strain. All strains harbor one N-acetylglucosamine 6-phosphate deacetylase belonging to the carbohydrate esterase of family 9 (CE9), while in the *W. diestrammenae* type strain, two additional CEs were annotated. Glycosyl transferases were the class with the highest count, with a total of 21 enzymes in the *W. ghanensis* type strain, 19 in the *W. fabaria* type strain, 16 in the *W. fabalis* type strain, 15 in the *W. diestrammenae* and *W. uvarum* type strains, and 14 in the *W. beninensis* type strain.

**Table 2:** Number of CAZymes present in sequenced *Weissella* strains.

	***W. beninensis* LMG 25373^**T**^**	***W. fabalis* LMG 26217^**T**^**	***W. uvarum* B18NM42^**T**^**	***W. fabaria* LMG 24289^**T**^**	***W. diestrammenae* DSM 27840^**T**^**	***W. ghanensis* DSM 19935^**T**^**
**Auxiliary Activities (AAs)**						
AA4	-	-	-	-	-	1
**Carbohydrate Binding-Module (CBMs)**						
CBM34	-	-	-	-	1	-
**Carbohydrate Esterases (CEs)**						
CE1	-	-	-	-	1	-
CE4	-	-	-	-	1	-
CE9	1	1	1	1	1	1
**Glycoside Hydrolases (GHs)**						
GH1	1	2	-	2	2	2
GH2	1	-	-	-	-	-
GH3	-	-	1	1	1	2
GH4	-	1	-	-	-	1
GH5_44	-	-	-	-	1	-
GH13_18	1	-	-	-	-	-
GH13_20	-	-	-	-	1	-
GH13_29	-	2	-	2	-	2
GH13_31	-	2	2	-	-	2
GH25	1	2	2	2	1	1
GH31	-	-	-	-	1	-
GH32	1	-	1	-	-	1
GH36	1	-	-	-	-	-
GH42	1	-	-	-	-	-
GH43_11	-	-	-	-	2	-
GH43_26	-	-	-	-	1	-
GH65	1	-	1	-	1	-
GH70	1	-	-	-	-	-
GH73	3	1	1	1	1	1
GH123	-	-	-	1	-	1
**Glycosyl Transferases (GTs)**						
GT2_Glyco_trans_2_3	1	-	-	-	-	-
GT2_Glycos_transf_2	4	4	4	5	6	7
GT4	4	8	4	10	5	10
GT8	-	-	1	-	-	-
GT28	1	1	1	1	1	1
GT32	-	1	-	1	-	1
GT51	2	2	4	2	2	2
GT83	1	-	-	-	-	-
GT111	1	-	1	-	1	-
**Polysaccharide Lyases (PLs)**						
PL1	-	1	-	1	-	-
PL8	-	1	-	1	-	1

**Figure 4:**
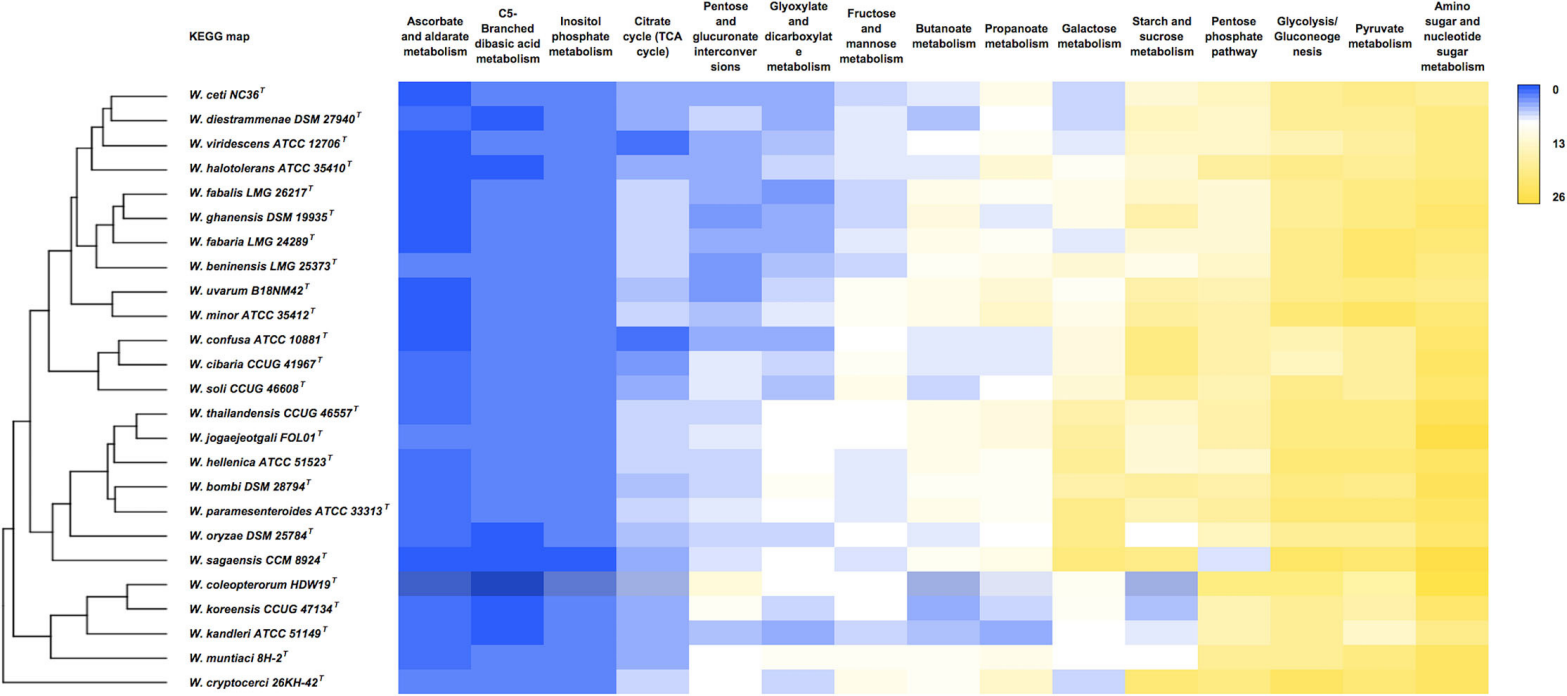
Comparative analysis of carbohydrate metabolism in *Weissella* species. The heatmap was manually constructed based on the number of proteins associated with each KEGG pathway in each genome and visualized with the average linkage as a clustering method and the Euclidean distance measurement method, providing the resulting dendrogram.

A comparative analysis of carbohydrate metabolism pathways is depicted in Figure 4. The analysis shows that the highest count of enzymes within these pathways was retrieved in the categories “amino sugar and nucleotide sugar metabolism,” “pyruvate metabolism,” “glycolysis and gluconeogenesis,” and “starch and glucose metabolism.” The *W. cryptocerci* type strain has the highest enzyme count (172 in total), while the lowest occurred in the *W. kandleri* type strain (111).

**Figure 5:**
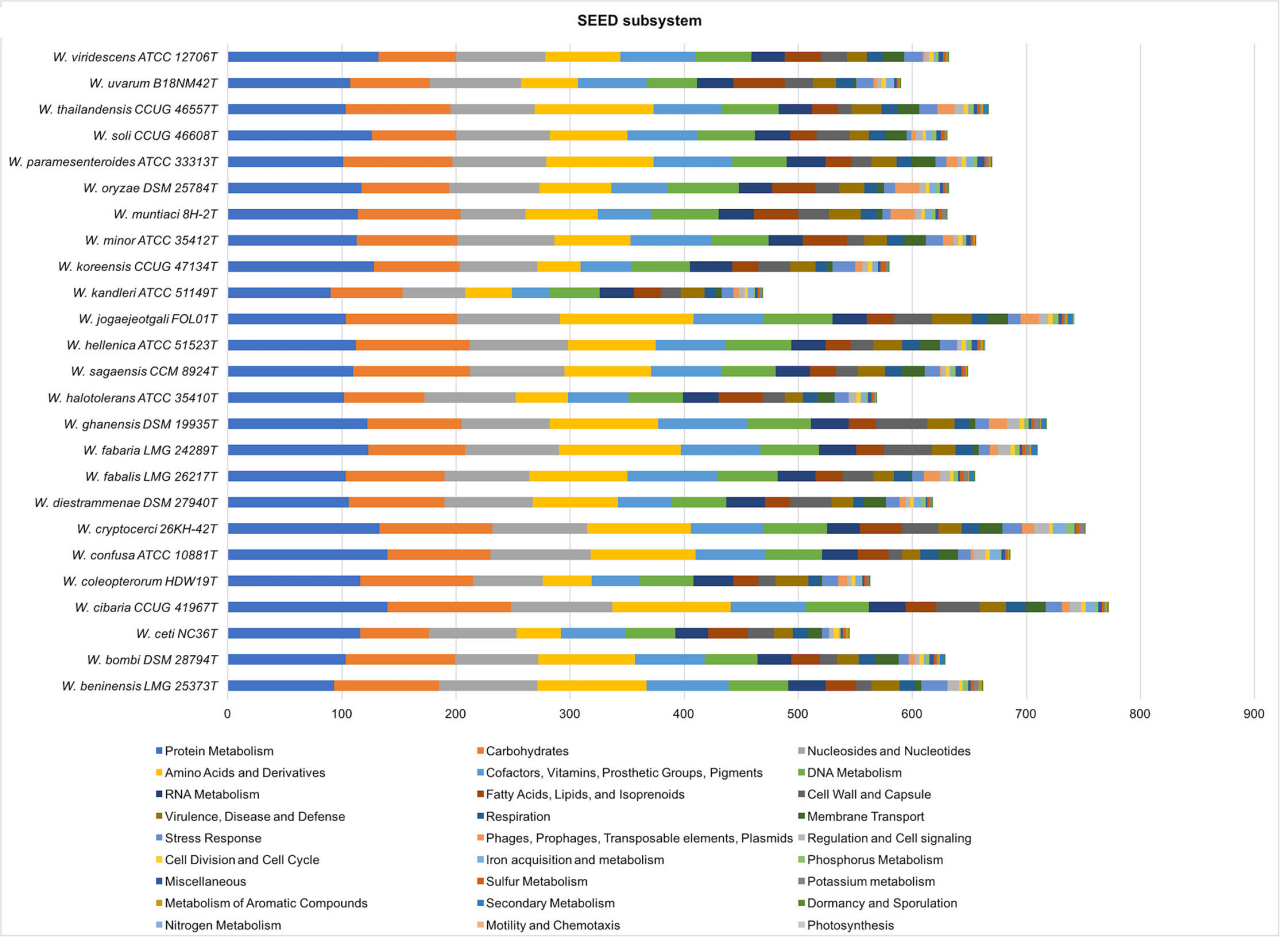
Comparative analysis of SEED subsystem features in *Weissella* species. Genes annotated by RAST were assigned to functional categories and grouped into subsystems. Colored bars indicate the number of genes assigned to each category.

A comparative analysis of the SEED subsystem is shown in Figure 5. The highest number of features was determined in the subsystem “protein metabolism,” followed by “carbohydrates and nucleosides and nucleotides.” The *W. cibaria, W. cryptocerci*, and *W. jogaejeotgali* type strains showed the highest total feature counts.

#### Substrate Consumption by *Weissella* Strains

Carbon source consumption was evaluated using Biolog AN plates, and the results are presented in Table 3. All *Weissella* strains used N-acetyl-D-glucosamine, D-fructose, α-D-glucose, D-mannose, palatinose, turanose, and maltotriose. Furthermore, according to the comparative analysis of carbohydrate metabolism pathways and the cluster analysis based on carbon source oxidation (Supplementary Figure S1), the cluster including *W. beninensis, W. fabalis, W. fabaria*, and *W. ghanensis* type strains was characterized by the consumption of glycyl-L-methionine, α-ketobutyric acid, and pyruvic acids. Only *W. fabalis* and *W. fabaria* consumed i-erythritol, D-trehalose, and D-malic acid (Table 3). Moreover, formic acid was used only by the *W. fabaria* type strain, while α-cyclodextrin, fumaric acid, and glycyl-L-glutamine were utilized by the *W. fabalis* type strain. Among all the strains tested, the *W. beninensis* type strain showed the widest consumption of carbon sources. In fact, only this strain was capable of using D-galactose, α-D-lactose, lactulose, D-melibiose, β-methyl-D-galactoside, D-raffinose, sucrose, pyruvic acid methyl ester, and uridine-5'-monophosphate. Conversely, D-arabitol and D-sorbitol were exclusively consumed by the *W. uvarum* type strain (Table 3).

**Table 3:** Consumption of carbon sources by *Weissella* strains evaluated after anaerobic incubation of Biolog AN MicroPlate (Biolog, USA).

	***W. beninensis* LMG 25373^**T**^**	***W. diestrammenae* DSM 27840^**T**^**	***W. fabalis* LMG 26217^**T**^**	***W. ghanensis* DSM 19935^**T**^**	***W. uvarum* B18NM42^**T**^**	***W. fabaria* LMG 24289^**T**^**
N-Acetyl-D-Glucosamine	+^a^	+	+	+	+	+
N-Acetyl-β-DMannosamine	–^b^	+	+	+	+	+
Amygdalin	–	–	+	+	–	–
D-Arabitol	–	–	–	–	+	–
D-Cellobiose	+	–	+	+	+	+
α-Cyclodextrin	–	–	+	–	–	–
Dextrin	+	–	+	+	+	+
i-Erythritol	–	–	+	–	–	+
D-Fructose	+	+	+	+	+	+
L-Fucose	+	–	–	+	–	–
D-Galactose	+	–	–	–	–	–
Gentiobiose	–	–	+	+	–	–
D-Gluconic Acid	+	+	–	+	+	–
α-D-Glucose	+	+	+	+	+	+
α-D-Glucose-1-Phosphate	–	+	–	–	+	–
D-Glucose-6-Phosphate	–	+	–	–	–	–
Glycerol	–	–	–	+	+	+
α-D-Lactose	+	–	–	–	–	–
Lactulose	+	–	–	–	–	–
Maltose	–	+	–	+	–	–
Maltotriose	+	+	+	+	+	+
D-Mannitol	+	–	+	–	+	+
D-Mannose	+	+	+	+	+	+
D-Melibiose	+	–	–	–	–	–
3-Methyl-D-Glucose	+	–	+	–	+	+
β-Methyl-D-Galactoside	+	–	–	–	–	–
β-Methyl-D-Glucoside	–	–	+	+	–	–
Palatinose	+	+	+	+	+	+
D-Raffinose	+	–	–	–	–	–
L-Rhamnose	+	–	+	+	–	–
Salicin	–	–	+	+	–	–
D-Sorbitol	–	–	–	–	+	–
Sucrose	+	–	–	–	–	–
D-Trehalose	–	–	+	–	–	+
Turanose	+	+	+	+	+	+
Acetic Acid	–	–	–	–	–	+
Formic Acid	–	–	–	–	–	+
Fumaric Acid	–	–	+	–	–	–
Glyoxylic Acid	–	–	+	+	–	+
α-Hydroxybutyric Acid	+	+	–	–	+	+
α-Ketobutyric Acid	+	+	+	+	–	+
α-Ketovaleric Acid	–	+	+	+	–	+
D,L-Lactic Acid	+	+	+	+	+	–
L-Lactic Acid	+	+	+	+	–	–
D-Lactic Acid Methyl Ester	+	+	+	–	+	+
D-Malic Acid	–	–	+	–	–	+
L-Malic Acid	+	+	+	+	–	–
Propionic Acid	–	–	–	+	–	+
Pyruvic Acid	+	–	+	+	+	+

*The Weisella strains resulted all negative for N-acetyl-D-galactosamine, adonitol, arbutin, β-cyclodextrin, dulcitol, D-galacturonic acid, D-glucosaminic acid, D,L-α-glycerol phosphate, m-inositol, D-melezitose, α-methyl-D-galactoside, α-methyl-D-glucoside, stachyose, β-hydroxybutyric acid, itaconic acid, D-saccharic acid, succinamic acid, succinic acid, succinic acid mono-methyl ester, m-tartaric acid, urocanic acid, alaninamide, L-alanine, L-alanyl-L-glutamine, L-alanyl-L-histidine, L-alanyl-L-threonine, L-asparagine, L-glutamic acid, L-glutamine, glycyl-L-aspartic acid, glycyl-L-proline, L-phenylalanine, L-serine, L-threonine, L-valine, L-valine plus L-aspartic acid, 2'-deoxy adenosine, thymidine, thymidine-5'-mono-phosphate*.

a *+, utilization/oxidation of the carbon source*;

b *-, not utilization/oxidation of the carbon source*.

## Discussion

Based on the results of ANI, AAI, 16S rRNA gene sequence identity, and 16S rRNA gene- and genome-based phylogeny, all *Weissella*-type strains investigated could be confirmed as representing individual and separate species. Based on our ANI/AAI results, we confirmed that *W. jogaejeotgali*, first described by Lee et al. (2015) as a novel species, could be classified as a later heterotypic synonym of *W. thailandensis*. Furthermore, we also confirmed that *W. kimchii*, described as a novel species by Choi et al. (2002), is a later heterotypic synonym of *W. cibaria*.

Our data partially validate the taxonomic clustering previously proposed by De Bruyne et al. (2010) and Fusco et al. (2015), which were essentially based on the 16S rRNA gene and *pheS* gene sequence phylogenies, with the latter showing a higher discriminatory power as a marker gene. The description of novel species (Lee et al., 2015; Praet et al., 2015; Heo et al., 2019; Li et al., 2020; Lin et al., 2020; Hyun et al., 2021; this study) and the availability of additional genomic sequences (refer to Supplementary Table S1) allowed us to perform a comprehensive genomic-based phylogenetic analysis of all species in the genus *Weissella*. Our analysis also integrates genomic indexes, which are currently considered as minimal standards to perform taxonomic classification. Therefore, based on our phylogenomic reconstruction, we were able to define six distinct species groups within the genus *Weissella*, i.e., the *W. beninensis, W. kandleri, W. confusa, W. halotolerans, W. oryzae*, and *W. paramesenteroides* species groups.

The first divergent line within the genus is constituted by the *W. beninensis* group, which includes *W. beninensis, W. cryptocerci, W. fabalis, W. fabaria*, and *W. ghanensis*. In this case, the clustering recently updated by Fusco et al. (2015) is confirmed by the genome-based phylogeny and by genomic analysis: the average ANI value among these species is 81.2%, with *W. ghanensis* and *W. fabaria* being the most closely related, with an ANI value of 85.63%. Species within this group share 96% of 16S rRNA gene sequence identity; the percentage rises to 99.5% among *W. fabaria, W. fabalis*, and *W. ghanensis*. The second species group, designated as the *W. kandleri* group, comprises *W. kandleri, W. diestrammenae, W. koreensis, W. soli*, and *W. coleoptorum*. The latter was described in 2021 (Hyun et al., 2021) and was therefore not represented in the previous classification. The 16S rRNA gene sequence identity among these species is 97.1% on average, while the ANI value is 82%, with *W. coleoptorum* and *W. soli* being the closest related, sharing an ANI value of 83.91%. In contrast to the results previously presented by Fusco et al. (2015), *W. oryzae* was not included in this group, as the genomic analysis performed in this study placed it into a separate branch together with *W. muntiaci*. These two species share 99.32% of 16S rRNA gene sequence identity, 80.15% of ANI, and 75.64 of AAI, and can be assigned to a third species group, namely the *W. oryzae* species group. The fourth species group, namely the *W. confusa* species group, comprises *W. confusa* and *W. cibaria*, confirming the clustering previously proposed by Fusco et al. (2015). Based on the results of this study, we added the recently described *W. sagaenisis* (Li et al., 2020) and *W. bombi* species (Praet et al., 2015) to the fifth species group, designated by Fusco et al. (2015) as *W. paramestenteroides*, which also includes *W. paramesenteroides, W. hellenica*, and *W. thailandensis*. The average ANI value shared among these species is 82.4%, while 16S rRNA gene sequence identity is 98%. Notably, *W. sagaenisis* and *W. hellenica* 16S rRNA gene sequences are identical, while their shared ANI value is 89.7%, thus confirming the correct designation of *W. sagaenisis* as a novel species. In accordance with Fusco et al. (2015), the sixth species group is the *W. halotolerans* group, which includes *W. halotolerans, W. ceti, W. uvarum, W. minor*, and *W. viridescens*. Within these species, the 16S rRNA gene sequence identity reaches 95.1%, with the maximum value of 99.14% occurring between *W. minor* and *W. uvarum*, while the shared ANI value for this group is 79.06%.

According to CAZy and KEGG pathway analyses, species within this genus harbor a comprehensive carbohydrate utilization system, including sugar uptake, transporters, and metabolism-related genes, which confer them strong carbohydrate utilization capabilities, as shown by different carbohydrate utilization profiles. The carbohydrate metabolism pathway clustering follows the phylogenomic species group clustering in many cases: this occurred for *W. beninensis, W. fabalis, W. fabaria*, and *W. ghanensis;* for *W. minor* and *W. uvarum*; for *W. ceti* and *W. halotolerans*; for *W. bombi* and *W. sagaensis*; and for *W. thailandensis, W. paramesenteroides*, and *W. jogaejeotgali*.

The *W. fabalis, W. ghanensis*, and *W. uvarum* type strains were found to harbor the genetic determinants for the synthesis of oligo-1,6-glucosidases, which hydrolyze the α-1,6 linkage in starch, glycogen, and the derived oligosaccharides to produce sugars with an α-configuration. The *W. diestrammenae* type strain was found to harbor a gene coding for a maltogenic amylase, an enzyme that favors starch degradation in maltose. Therefore, these *Weissellas* may play an important role in the fermentation of sourdoughs due to the metabolism of starch and amylopectin, which are composed of α-1,4 glucose main chain and α-1,6 glucose side chain (van der Maarel et al., 2002; Wang et al., 2021).

All strains were able to use the D-mannose sugar. This sugar is a dextrorotatory hexose aldehyde/aldose monosaccharide found in certain bacteria, fungi, and plants, and is rarely present in nature as a free monosaccharide. Nevertheless, it is a constituent of numerous simple and complex polysaccharides and is mostly found in nature as a component of mannan, hemicellulose, and cellulose in dietary fiber (Hu et al., 2016). For example, it constitutes the basic molecule of mannans, the reserve polysaccharides of some plant species (e.g., palm), or is associated with galactose (mannogalactans) to form gummy mucilages that protect the seeds of some plants (e.g., carob); they are widely used as stabilizers of food products such as ice cream and mayonnaise. The *W. diestrammenae* type strain was isolated from the gut of a camel cricket. The remaining 5 type strains sequenced in this study originated from vegetables such as cassava, cocoa, and grapes, all sources containing mannose and/or its (oligo)polymers. Due to the described enzymatic activities, these *Weissellas* could be employed as starters for the fermentation of such mannose- and oligo mannose-containing vegetables. Their ability to metabolize mannose is corroborated by the fact that in every sequenced genome we retrieved (i) the *man*Xa transporter, which can transfer phosphorus-containing groups to D-mannose, and (ii) the mannose-6-phosphate isomerase [EC:5.3.1.8] for the conversion of D-mannose-6-phosphate to β-D-fructose 6-phosphate. The mannose transportation operon *man*XYZ (Jeckelmann and Erni, 2020) was identified in all the sequenced strains.

Except for *W. diestrammenae*, all the other strains possess an *scr*K gene, coding for a fructokinase. It catalyzes the transfer of phosphate to D-fructose, which is then converted to α-D-glucose 6-phosphate by the glucose-6-phosphate isomerase. In the *W. diestrammaneae* type strain, fructose utilization might proceed through its conversion to α-D-glucose, catalyzed by the xylose isomerase, only harbored by this strain (*xyl*A gene, KAR27_02655). In the *W. uvarum* and *W. fabaria* type strains, the *fru*AB gene, coding for the D-fructose phosphotransferase, was identified. Only in the *W. uvarum* type strain can D-fructose enter glycolysis through the subsequent action of the *fru*K fructokinase and the triosephosphate isomerase, which converts the β-D-fructose 1,6-bisphosphate to glyceraldehyde 3-phosphate.

According to the Biolog data, *W. beninensis* is the only species able to metabolize D-galactose and raffinose. In fact, the *gal*A gene was detected only in the genomic sequence of the type strain of this species, in addition to two copies of the β-galactosidase (KAK10_01750 and KAK10_05755). The *gal*A codes for the α-galactosidase [EC:3.2.1.22] (KAK10_01740), the exoglycosidase that hydrolyzes α-1,6 galactoside linkages found in sugars, such as raffinose, melibiose, and stachyose, and branched polysaccharides such as galactomannans and galacto-glucomannans. The ability to metabolize nondigestible sugars such as oligosaccharides of the raffinose family and galactomannans is common for several probiotic bacteria (Zartl et al., 2018). The α-galactosidase enzyme is well explored in the food industry, where it is used for removing raffinose family oligosaccharides (RFOs) in soymilk and sugar crystallization processes, and for the improvement of animal feed quality and biomass processing (Bhatia et al., 2020).

Only *W. beninensis* can consume D-sucrose. This is due to the presence of (i) a PTS transporter subunit EIIC **(**KAK10_07455), which transports sucrose into the cell, converting it to sucrose-6-phosphate; (ii) a sucrose-6-phosphate hydrolase (KAK10_07450), which catalyzes the formation of fructose and glucose-6-phosphate; and (iii) a sucrose phosphorylase (KAK10_07625), which reconverts the sucrose-6-phosphate into sucrose. Moreover, a preliminary evaluation of EPS production (data not shown) indicates that *W. beninensis* can produce ropiness when incubated in MRS added with 20 g/L of sucrose. *W. beninensis* was isolated from spontaneous fermentation of cassava, and it was previously characterized for the acid production from D-fructose, D-galactose, D-glucose, lactose, maltose, D-mannose, melibiose, D-raffinose, sucrose, *N*-acetylglucosamine, and D-mannitol (Padonou et al., 2010). Cassava usually contains a low amount of free sugar, which differs according to the variety and the tissue age in the storage root (Junior and Campos, 2004). Its spontaneous fermentation is accomplished by the presence of different species of yeasts and lactic acid bacteria (Padonou et al., 2009) and activates mutually stimulating interactions, as widely characterized in other products such as sourdough and kefir (De Vuyst and Neysens, 2005; Tofalo et al., 2020). The ability of *W. beninensis* to use numerous carbon sources may indicate its adaptation to this complex microbial and metabolic environment.

**Figure 6:**
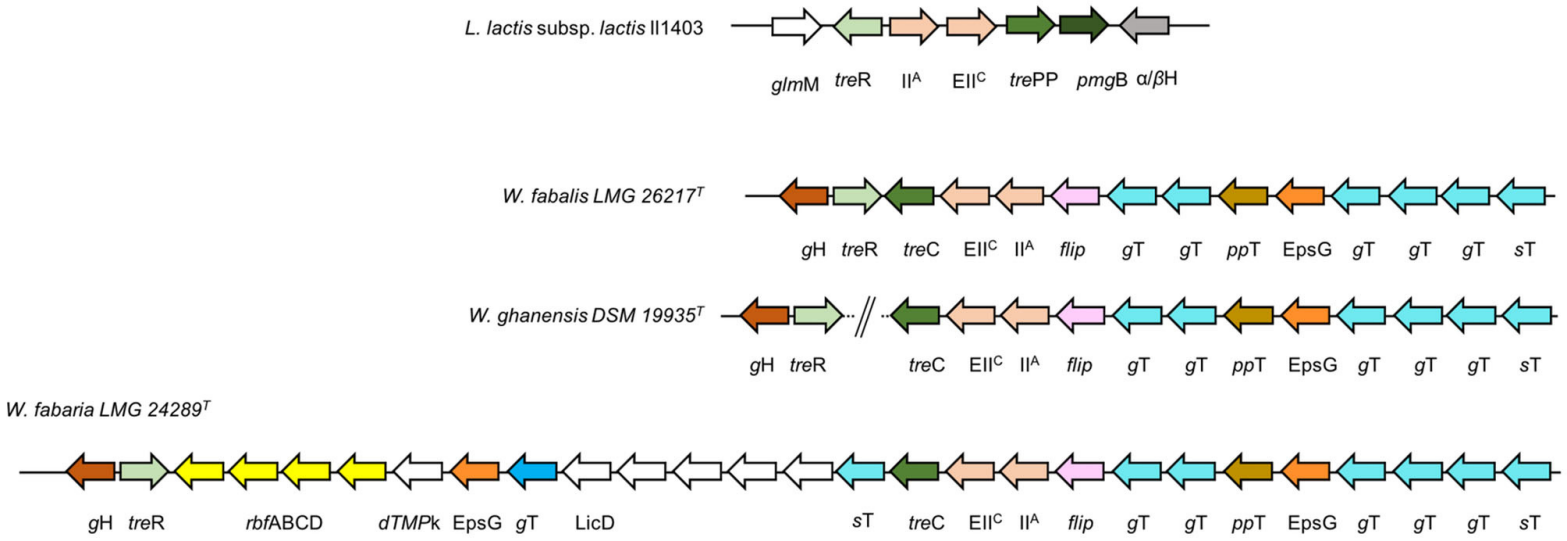
Trehalose operon in *Weissella* species. Genomic organization of the putative trehalose operon in the *Weissella* species. Gene clustering is represented by the arrows superposed on the black horizontal line. Gene and intergenic spaces are not drawn in scale. *Lactococcus lactis* subsp. *lactis* Il1403 (GenBank accession no. NC_002662): *glm*M, phosphoglucosamine mutase; *tre*R, trehalose operon repressor; IIA, PTS glucose transporter subunit IIA; EIIc, PTS transporter subunit EIIC; *pmg*B, beta-phosphoglucomutase; α/βH, alpha/beta hydrolase; gH, glycosyl-hydrolase; polysaccharide biosynthesis C-terminal domain-containing protein flip, flippase; *g*T, glycosyltransferase; *pp*T, polysaccharide pyruvyl transferase family protein; EpsG, EpsG family protein; *s*T, sugar transferase; *rbf*ABCD, rhamnose operon; dTMPk, dTMP kinase, LicD, LicD family protein.

Trehalose, or α-D-glucopyranosyl-1,1-α-D-glucopyranoside, is a disaccharide made up of two α-D-glucose molecules joined by an α-1,1 glycosidic bond. It naturally occurs in bacteria, fungi, yeasts, algae, plants, and invertebrates, including insects, but it is absent in vertebrates. Its major dietary sources are mushrooms; in fact, it is contained in most edible fungi and is an important part of reconstituting dried shiitake mushrooms. For this reason, it is also referred to as a mushroom sugar, while it is called seaweed sugar in China since trehalose is contained in marine plants such as “hijiki” seaweed. *Weissellas* that can metabolize this sugar may play a pivotal role in the fermentation and digestion of the above-mentioned (novel) foods. To metabolize trehalose, this disaccharide enters the cells throughout the action of the trehalose PTS permease (EC: 2.7.1.201). Consequently, the α,α-phosphotrehalase catalyzes the hydrolysis of α,α-trehalose 6-phosphate into D-glucose and D-glucose 6-phosphate. This enzyme was annotated in the genomic sequences of the *W. fabalis, W. fabaria*, and *W. ghanensis* type strains to occur in a genomic cluster, which shows differences in the organization among these species (Figure 6), while the corresponding genes in the *W. ghanensis* type strain were annotated in two separate contigs. All these 3 species share the same ecological niche of origin (*W. ghanensis* type strain was isolated from Ghanaian cocoa fermentation; *W. fabaria* type strain was isolated from fermented cocoa bean heaps; and *W. fabalis* was isolated from spontaneous cocoa bean fermentation). Common to all 3, within the cluster, there are (1) a gene coding for a hypothetical protein with a GH (family 13) catalytic domain, (2) the *tre*R gene (coding for the trehalose operon repressor), (3) the *tre*C gene (coding for the α,α-phosphotrehalase), (4) the genes coding for TreB (the trehalose transporter with the two domain phosphotransferases (PTS) subunit EIIC and IIA), (5) one flippase gene, (6) one gene coding for an EpsG family protein, (7) one for a sugar transferase, and (8) several genes coding for glycosyl transferases. In the *W. fabaria* type strain, this locus also comprises a sequence of 14 kbp downstream of *tre*R, where there are annotated rhamnose operon *rbf* ABCD and genes coding for (1) a dTMP kinase, (2) a LicD family protein, (3) a DUF1972 domain-containing protein, (4) a CpsD/CapB family tyrosine-protein kinase, (5) a capsular biosynthesis protein, (6) an LCP family protein, and (7) a tyrosine phosphatase. The trehalose operon has not yet been described in the *Weissella* species. In addition to the *tre*R and *tre*B genes, *Lactococcus lactis* includes the phosphomannomutase gene *fem*B, the trehalose/maltose hydrolase gene *tre*PP, and the β-phosphoglucomutase *pgm*B gene (Andersson et al., 2005) (Figure 6). The *pgm*B gene was instead annotated in *W. diestrammenae* (KAR27_07555), within a locus comprising genes coding for the transcriptional regulator LacI, an α-glycosidase, a glycoside hydrolase, a galactose maturotase, and sugar transporters.

The maltose operon was first described in *L. lactis* by Andersson and Rådström (2002). Maltose is transported by an ATP-dependent permease system, and then it is degraded by the concerted action of a Pi-dependent maltose phosphorylase (MP) and the β-phosphoglucomutase (β-PGM), which are located in two different operons. In the *Weissella* species analyzed in this study, *pgm*B occurred in the *W. diestrammenae* (KAR27_07555), *W. beninensis* (KAK10_00105), and *W. uvarum* (KAR63_02130) type strains. The *map*A gene, which codes for the maltose phosphorylase [EC:2.4.1.8] that catalyzes the reaction between maltose and phosphate to form D-glucose and β-D-glucose-1-phosphate, was found to be present in the *W. uvarum* (KAR63_02125), *W. beninensis* (KAK10_00115), and *W. diestrammenae* (KAR27_07545) type strains. In contrast to *L. lactis*, the *mal*P and *pgm*B genes are located closely on the genomic sequences of the species in which they were annotated. In the *W. uvarum* type strain, this operon also contains genes coding for the glucose transport system, formed by the PTS transporter subunit EIIC and PTS glucose transporter subunit IIA, and a trehalose operon repressor. However, the sequences surrounding this genomic area are different from the trehalose operon as above described in the other *Weissella* species, indicating a divergent evolutionary pattern. In the *pgm*B genomic locus of the *W. diestrammenae* type strain, there are also genes coding for a galactose mutarotase, a glycosyl hydrolase, the α-glycosidase (KAR27_07540) gene, a gene coding for an MFS transporter, one for a LacI family DNA-binding transcriptional regulator, one for an ECF transporter, one for an ABC transporter substrate-binding protein, and a permease gene. The *pgm*B gene codes for a β-phosphoglucomutase, which catalyzes the interconversion of D-glucose 1-phosphate (G1P) and D-glucose-6-phosphate (G6P), forming β-D-glucose 1,6-(bis)phosphate (β-G16P) as an intermediate. It is the catabolic activity in the maltose and trehalose pathways. Although *pgm*B has not yet been characterized in the *Weissella* species, this gene was reported as essential in the trehalose pathway of *L. lactis* (Andersson et al., 2001). The occurrence of this gene in the *W. diestrammenae* type strain, which, according to the carbon source consumption analysis, is not able to metabolize trehalose, suggests its involvement in the maltose metabolism. This sugar is indeed consumed by this strain, most likely through the subsequent reactions catalyzed by the maltose phosphorylase MapA and PgmB. In the *W. beninensis* type strain, the putative maltose operon was annotated in a very short contig; therefore, it was not possible to reconstruct the surrounding organization of the putative operon, although there are annotated genes coding for a galactose mutarotase, a glycosyl hydrolase, one MFS transporter, and two LacI family DNA-binding transcriptional regulators. In the W. *fabalis, W. uvarum*, and *W. ghanensis* type strains, the ability to hydrolyze maltose can also be associated with the presence of the maltase-glucoamylase (E. 3.2.10) that catalyzes maltose hydrolysis to produce two molecules of D-glucose.

Furthermore, α-glucosidase genes were retrieved in the *W. fabalis, W. ghanensis*, and *W. uvarum* type strains. The substrates for this hydrolytic enzyme are maltooligosaccharides, phenyl α-maltoside, nigerose, soluble starch, amylose, amylopectin, and β-limit dextrins (Tomasik and Horton, 2012). In a recent study by Wangpaiboon et al. (2021), this enzyme was characterized in *W. cibaria* as acting on short-chain maltooligosaccharides. Genes encoding 6-phospho-β-glucosidase enzymes were annotated in the *W. diestrammenae, W. fabalis, W. fabaria*, and *W. ghanensis* type strains. This enzyme is involved in the hydrolysis of phosphorylated disaccharides and usually does not have hydrolytic activity toward nonphosphorylated substrates. The resulting glucose and glucose 6-phosphate are further metabolized by the glycolytic pathway. β-D-glucosidase activity is widespread among lactic acid bacteria. It was suggested to play a role in the interaction with the human host. Furthermore, it is relevant for food fermentation processes and is involved in the release of β-glucosidases from their β-D-glucosylated precursors in several plant secondary metabolites (Michlmayr and Kneifel, 2014).

In *W. beninensis*, only one GH1 was annotated, in the putative ribose operon, comprising sugar transporters, and the *rbsC* and *rbs*D genes, coding for the ribose permease and the D-ribose pyranase, respectively.

Pectin lyases were identified in the genomic sequences of the *W. fabalis, W. fabaria*, and *W. ghanensis* type strains. These enzymes catalyze pectin degradation *via* eliminative cleavage of the α-(1,4) glycosidic linkages in homogalacturonan. The capability of these species to hydrolyze pectin suggested by the presence of these enzymes could be relevant for the exploitation in the food industry; pectin lyases are, in fact, usually employed in wine and juice prepress maceration, as well as in juice clarification methods (Mantovani et al., 2005; Kassara et al., 2019).

## Conclusion

In this study, for the first time, we sequenced and analyzed the genomes of the type strains of 6 *Weissella* species discovered in the past decade, whose assemblies were not yet available at the time this study was initiated. We also performed a comprehensive phylogenomic analysis on all the *Weissella* species to date described and reassessed the phylogenetic structure of strains in the *Weissella* genus using 16S rRNA gene sequence and genome-based phylogeny. Updating the previous clustering proposed by other authors, we were able to identify six distinct species groups within the genus, namely, *W. beninensis, W. kandleri, W. confusa, W. halotolerans, W. oryzae*, and *W. paramesenteroides* species groups.

Moreover, we investigated the capability of the 6 type strains to metabolize 95 carbohydrates, demonstrating the strong carbohydrate utilization capabilities of the sequenced strains. This ability was also confirmed by the identification of the genetic determinants of the enzymes involved in carbohydrate metabolism. The genomic and phenotypic analyses provided further knowledge about the ability of the *W. beninensis, W. ghanensis, W. fabaria, W. fabalis, W. uvarum*, and *W. diestrammenae* type strains to metabolize certain carbohydrates and to detect their genetic determinants. In fact, the studies reporting the discovery of these type strains (De Bruyne et al., 2008, 2010; Padonou et al., 2010; Oh et al., 2013; Snauwaert et al., 2013; Nisiotou et al., 2014) provided data about the utilization of a maximum of 19 carbohydrates against the 95 carbohydrates tested herein by using the Biolog. Furthermore, the permutation analysis of the Biolog data confirmed the interspecific metabolic diversity of the analyzed type strains.

The increasing availability of the genomic sequences of the *Weissella* species will contribute to improving the knowledge about this genus and identifying the features defining its role in fermentative processes and its biotechnological potential.

## Data Availability Statement

The datasets presented in this study can be found in online repositories. The names of the repository/repositories and accession number(s) can be found in the article/Supplementary Material.

## Author Contributions

VF conceived the work and interpreted the data. G-SC performed the genomic sequencing. VF and FF organized and performed the bioinformatic work. DC checked the purity of the strains and prepared the working cultures for DNA extraction and phenotypic characterization. MM performed the phenotypic characterization. VF, FF, and MM wrote the manuscript. All authors contributed to the revision of the manuscript, read, and approved the submitted version.

## Conflict of Interest

The authors declare that the research was conducted in the absence of any commercial or financial relationships that could be construed as a potential conflict of interest.

## Publisher's Note

All claims expressed in this article are solely those of the authors and do not necessarily represent those of their affiliated organizations, or those of the publisher, the editors and the reviewers. Any product that may be evaluated in this article, or claim that may be made by its manufacturer, is not guaranteed or endorsed by the publisher.

## Supplementary Material

Supplementary Table S1 List of *Weissella* strains used in this study.

Supplementary Table S2 ANI values among *Weissella* strains.

Supplementary Table S3 AAI values among *Weissella* strains.

Supplementary Table S4 CAZymes present in sequenced *Weissella* species.

Supplementary Figure S1 Cluster analysis of *Weissella* strains based on carbon source consumption by PermutMatrix.

## References

[B1] Afgan E. Baker D. Batut B. van den Beek M. Bouvier D. Cech M.. (2018). The Galaxy platform for accessible, reproducible and collaborative biomedical analyses: 2018 update. Nucleic Acids Res. 46, W537–W544. 10.1093/nar/gky379 29790989 PMC6030816

[B2] Afolayan A. O. Ayeni F. A. Ruppitsch W. (2017). Antagonistic and quantitative assessment of indigenous lactic acid bacteria in different varieties of *ogi* against gastrointestinal pathogens. Pan Afr. Med. J. 27, 22. 10.11604/pamj.2017.27.22.9707 28748023 PMC5511708

[B3] Albesharat R. Ehrmann M. A. Korakli M. Yazaji S. Vogel R. F. (2011). Phenotypic and genotypic analyses of lactic acid bacteria in local fermented food, breast milk and faeces of mothers and their babies. Syst. Appl. Microbiol. 34, 148–155. 10.1016/j.syapm.2010.12.001 21300508

[B4] Andersson U. Levander F. Rådström P. (2001). Trehalose-6-phosphate phosphorylase is part of a novel metabolic pathway for trehalose utilization in *Lactococcus lactis*. J. Biol. Chem. 276, 42707–42713. 10.1074/jbc.M108279200 11553642

[B5] Andersson U. Molenaar D. Rådström P. de Vos W. M. (2005). Unity in organisation and regulation of catabolic operons in *Lactobacillus plantarum*, L*actococcus lactis* and *Listeria monocytogenes*. Syst. Appl. Microbiol. 28, 187–195. 10.1016/j.syapm.2004.11.004 15900965

[B6] Andersson U. Rådström P. (2002). Physiological function of the maltose operon regulator, MalR, in *Lactococcus lactis*. BMC Microbiol. 2, 28. 10.1186/1471-2180-2-28 12296976 PMC130022

[B7] Aziz R. K. Bartels D. Best A. A. DeJongh M. Disz T. Edwards R. A.. (2008). The RAST Server: rapid annotations using subsystems technology. BMC Genom. 9, 75. 10.1186/1471-2164-9-75 18261238 PMC2265698

[B8] Babicki S. Arndt D. Marcu A. Liang Y. Grant J. R. Maciejewski A.. (2016). Heatmapper: web-enabled heat mapping for all. Nucleic Acids Res. 44, W147–W153. 10.1093/nar/gkw419 27190236 PMC4987948

[B9] Bankevich A. Nurk S. Antipov D. Gurevich A. A. Dvorkin M. Kulikov A. S.. (2012). SPAdes: a new genome assembly algorithm and its applications to single-cell sequencing. J. Comput. Biol. 19, 455–477. 10.1089/cmb.2012.0021 22506599 PMC3342519

[B10] Beasley S. S. Manninen T. J. Saris P. E. (2006). Lactic acid bacteria isolated from canine faeces. J. Appl. Microbiol. 101, 131–138. 10.1111/j.1365-2672.2006.02884.x 16834600

[B11] Bhatia S. Singh A. Batra N. Singh J. (2020). Microbial production and biotechnological applications of α-galactosidase. Int. J. Biol. Macromol. 150, 1294–1313. 10.1016/j.ijbiomac.2019.10.140 31747573

[B12] Björkroth K. J. Schillinger U. Geisen R. Weiss N. Hoste B. Holzapfel W. H.. (2002). Taxonomic study of *Weissella confusa* and description of *Weissella cibaria* sp. nov., detected in food and clinical samples. Int. J. Syst. Evol. Microbiol. 52(Pt 1), 141–148. 10.1099/00207713-52-1-141 11837296

[B13] Bolger A. M. Lohse M. Usadel B. (2014). Trimmomatic: a flexible trimmer for Illumina sequence data. Bioinformatics 30, 2114–2120. 10.1093/bioinformatics/btu170 24695404 PMC4103590

[B14] Cai Y. Benno Y. Nakase T. Oh T. K. (1998). Specific probiotic characterization of *Weissella hellenica* DS-12 isolated from flounder intestine. J. Gen. Appl. Microbiol. 44, 311–316. 10.2323/jgam.44.311 12501410

[B15] Chen Y. S. Yanagida F. Shinohara T. (2005). Isolation and identification of lactic acid bacteria from soil using an enrichment procedure. Lett. Appl. Microbiol. 40, 195–200. 10.1111/j.1472-765X.2005.01653.x 15715644

[B16] Choi A. R. Patra J. K. Kim W. J. Kang S. S. (2018). Antagonistic activities and probiotic potential of lactic acid bacteria derived from a plant-based fermented food. Front. Microbiol. 9, 1963. 10.3389/fmicb.2018.01963 30197633 PMC6117381

[B17] Choi H. J. Cheigh C. I. Kim S. B. Lee J. C. Lee D. W. Choi S. W.. (2002). *Weissella kimchii* sp. nov., a novel lactic acid bacterium from kimchi. Int. J. Syst. Evol. Microbiol. 52(Pt 2), 507–511. 10.1099/00207713-52-2-507 11931163

[B18] Collins M. D. Samelis J. Metaxopoulos J. Wallbanks S. (1993). Taxonomic studies on some Leuconostoc-like organisms from fermented sausages: description of a new genus *Weissella* for the *Leuconostoc paramesenteroides* group of species. J. Appl. Bacteriol. 75, 595–603. 10.1111/j.1365-2672.1993.tb01600.x 8294308

[B19] De Bruyne K. Camu N. De Vuyst L. Vandamme P. (2010). *Weissella fabaria* sp. nov., from a *Ghanaian cocoa* fermentation. Int. J. Syst. Evol. Microbiol. 60, 1999-−2005. 10.1099/ijs.0.019323-0 19801391

[B20] De Bruyne K. Camu N. Lefebvre K. De Vuyst L. Vandamme P. (2008). *Weissella ghanensis* sp. nov., isolated from a Ghanaian cocoa fermentation. Int. J. Syst. Evol. Microbiol. 58(Pt 12), 2721–2725. 10.1099/ijs.0.65853-0 19060047

[B21] De Vuyst L. Neysens P. (2005). The sourdough microflora: biodiversity and metabolic interactions. Trends Food Sci. Technol. 16, 43–56. 10.1016/j.tifs.2004.02.012

[B22] Dereeper A. Guignon V. Blanc G. Audic S. Buffet S. Chevenet F.. (2008). Phylogeny.fr: robust phylogenetic analysis for the non-specialist. Nucleic Acids Res. 36, W465–W469. 10.1093/nar/gkn180 18424797 PMC2447785

[B23] Emerenini E. C. Afolabi O. R. Okolie P. I. Akintokun A. K. (2014). *In vitro* studies on antimicrobial activities of lactic acid bacteria isolated from fresh vegetables for biocontrol of tomato pathogens. Brit. Microbiol. Res. J. 4, 351–359. 10.9734/BMRJ/2014/5423

[B24] Ennahar S. Cai Y. (2004). Genetic evidence that *Weissella kimchii* Choi et al., 2002 is a later heterotypic synonym of *Weissella cibaria* Björkroth et al., 2002. Int. J. Syst. Evol. Microbiol. 54(Pt 2), 463–465. 10.1099/ijs.0.02783-0 15023961

[B25] Espinoza-Monje M. Campos J. Alvarez Villamil E. Jerez A. Dentice Maidana S. Elean M.. (2021). Characterization of *Weissella viridescens* UCO-SMC3 as a potential probiotic for the skin: its beneficial role in the pathogenesis of acne vulgaris. Microorganisms 9, 1486. 10.3390/microorganisms9071486 34361921 PMC8307422

[B26] Fanelli F. Chieffi D. Di Pinto A. Mottola A. Baruzzi F. Fusco V. (2020). Phenotype and genomic background of *Arcobacter butzleri* strains and taxogenomic assessment of the species. Food Microbiol. 89, 103416. 10.1016/j.fm.2020.103416 32138986

[B27] Fusco V. Quero G. M. Cho G. S. Kabisch J. Meske D. Neve H.. (2015). The genus *Weissella*: taxonomy, ecology and biotechnological potential. Front. Microbiol. 6, 155. 10.3389/fmicb.2015.00155 25852652 PMC4362408

[B28] Fusco V. Quero G. M. Stea G. Morea M. Visconti A. (2011). Novel PCR-based identification of *Weissella confusa* using an AFLP-derived marker. Int. J. Food Microbiol. 145, 437–443. 10.1016/j.ijfoodmicro.2011.01.015 21296447

[B29] Galle S. Schwab C. Arendt E. Gänzle M. (2010). Exopolysaccharide-forming *Weissella* strains as starter cultures for sorghum and wheat sourdoughs. J. Agric. Food Chem. 58, 5834–5841. 10.1021/jf1002683 20405917

[B30] Galli V. Venturi M. Coda R. Maina N. H. Granchi L. (2020). Isolation and characterization of indigenous *Weissella confusa* for in situ bacterial exopolysaccharides (EPS) production in chickpea sourdough. Food Res. Int. 138(Pt B), 109785. 10.1016/j.foodres.2020.109785 33288171

[B31] Goris J. Konstantinidis K. T. Klappenbach J. A. Coenye T. Vandamme P. Tiedje J. M. (2007). DNA-DNA hybridization values and their relationship to whole-genome sequence similarities. Int. J. Syst. Evol. Microbiol. 57, 81–91. 10.1099/ijs.0.64483-0 17220447

[B32] Heo J. Hamada M. Cho H. Weon H. Y. Kim J. S. Hong S. B.. (2019). *Weissella cryptocerci* sp. nov., isolated from gut of the insect *Cryptocercus kyebangensis*. Int. J. Syst. Evol. Microbiol. 69, 2801–2806. 10.1099/ijsem.0.003564 31246166

[B33] Hu X. Shi Y. Zhang P. Miao M. Zhang T. Jiang B. (2016). D-Mannose: properties, production, and applications: an overview. Comp. Rev. Food Sci. Food Saf. 15, 773–785. 10.1111/1541-4337.12211 33401842

[B34] Hyun D. W. Lee J. Y. Sung H. Kim P. S. Jeong Y. S. Lee J. Y.. (2021). *Brevilactibacter coleopterorum* sp. nov., isolated from the intestine of the dark diving beetle *Hydrophilus acuminatus*, and *Weissella coleopterorum* sp. nov., isolated from the intestine of the diving beetle *Cybister lewisianus*. Int. J. Syst. Evol. Microbiol. 71, 4779 10.1099/ijsem.0.004779 33886445

[B35] Jeckelmann J. M. Erni B. (2020). The mannose phosphotransferase system (Man-PTS) - Mannose transporter and receptor for bacteriocins and bacteriophages. Biochim. Biophys. Acta Biomembr. 1862, 183412. 10.1016/j.bbamem.2020.183412 32710850

[B36] Junior C. B. Campos L. (2004). Identification and characterization of a novel cassava (Manihot esculenta Crantz) clone with high free sugar content and novel starch. Plant Mol. Biol. 56, 643–659. 10.1007/s11103-004-4873-9 15630625

[B37] Kanehisa M. Sato Y. (2020). KEGG Mapper for inferring cellular functions from protein sequences. Protein Sci. 29, 28–35. 10.1002/pro.3711 31423653 PMC6933857

[B38] Kassara S. Li S. Smith P. Blando F. Bindon K. (2019). Pectolytic enzyme reduces the concentration of colloidal particles in wine due to changes in polysaccharide structure and aggregation properties. Int. J. Biol. Macromol. 140, 546–555. 10.1016/j.ijbiomac.2019.08.043 31404601

[B39] Kwak M. J. Choi S. B. Kim B. Y. Chun J. (2019). Genome-based reclassification of *Weissella jogaejeotgali* as a later heterotypic synonym of *Weissella thailandensis*. Int. J. Syst. Evol. Microbiol. 69, 3672–3675. 10.1099/ijsem.0.003315 31663499

[B40] Lee K. W. Park J. Y. Jeong H. R. Heo H. J. Han N. S. Kim J. H. (2012). Probiotic properties of *Weissella* strains isolated from human faeces. Anaerobe 18, 96–102. 10.1016/j.anaerobe.2011.12.015 22200451

[B41] Lee S. H. Ku H. J. Ahn M. J. Hong J. S. Lee S. H. Shin H.. (2015). *Weissella jogaejeotgali* sp. *nov.*, isolated from jogae jeotgal, a traditional Korean fermented seafood. Int. J. Syst. Evol. Microbiol. 65, 4674–4681. 10.1099/ijsem.0.000631 26410078

[B42] Letunic I. Bork P. (2019). Interactive Tree Of Life (iTOL) v4: recent updates and new developments. Nucleic Acids Res. 47, W256–W259. 10.1093/nar/gkz239 30931475 PMC6602468

[B43] Li J. Ai L. Xu F. Hu X. Yao Y. Wang L. (2021). Structural characterization of exopolysaccharides from *Weissella cibaria* NC516.11 in distiller grains and its improvement in gluten-free dough. Int. J. Biol. Macromol. 199, 17–23. 10.1016/j.ijbiomac.2021.12.089 34952097

[B44] Li Y. Q. Tian W. L. Gu C. T. (2020). *Weissella sagaensis* sp. nov., isolated from traditional Chinese yogurt. Int. J. Syst. Evol. Microbiol. 70, 2485–2492. 10.1099/ijsem.0.004062 32100692

[B45] Lin S. T. Wang L. T. Wu Y. C. Guu J. J. Tamura T. Mori K.. (2020). *Weissella muntiaci* sp. nov., isolated from faeces of Formosan barking deer (*Muntiacus reevesi*). Int. J. Syst. Evol. Microbiol. 70, 1578–1584. 10.1099/ijsem.0.003937 32228749

[B46] Mantovani C. F. Geimba M. P. Brandelli A. (2005). Enzymatic clarification of fruit juices by fungal pectin lyase. Food Biotechnol. 19, 173–181. 10.1080/0890543050031628428600206

[B47] Martín R. Heilig H. G. Zoetendal E. G. Jiménez E. Fernández L. Smidt H.. (2007). Cultivation-independent assessment of the bacterial diversity of breast milk among healthy women. Res. Microbiol. 158, 31–37. 10.1016/j.resmic.2006.11.004 17224259

[B48] Masuda Y. Zendo T. Sawa N. Perez R. H. Nakayama J. Sonomoto K. (2012). Characterization and identification of weissellicin Y and weissellicin M, novel bacteriocins produced by *Weissella hellenica* QU 13. J. Appl. Microbiol. 112, 99–108. 10.1111/j.1365-2672.2011.05180.x 22008177

[B49] Michlmayr H. Kneifel W. (2014). β-Glucosidase activities of lactic acid bacteria: mechanisms, impact on fermented food and human health. FEMS Microbiol. Lett. 352, 1–10. 10.1111/1574-6968.12348 24330034

[B50] Montemurro M. Pontonio E. Rizzello C. G. (2021). Design of a “Clean-Label” gluten-free bread to meet consumers demand. Foods 10, 462. 10.3390/foods10020462 33672491 PMC7923426

[B51] Mortezaei F. Royan M. Allaf Noveirian H. Babakhani A. Alaie Kordghashlaghi H. Balcázar J. L. (2020). *In vitro* assessment of potential probiotic characteristics of indigenous *Lactococcus lactis* and *Weissella oryzae* isolates from rainbow trout (*Oncorhynchus mykiss* Walbaum). J. Appl. Microbiol. 129, 1004–1019. 10.1111/jam.14652 32248610

[B52] Mun S. Y. Chang H. C. (2020). Characterization of *Weissella koreensis* SK isolated from kimchi fermented at low temperature (around 0 °c) based on complete genome sequence and corresponding phenotype. Microorganisms 8, 1147. 10.3390/microorganisms8081147 32751267 PMC7464874

[B53] Muñoz-Atienza E. Gómez-Sala B. Araújo C. Campanero C. del Campo R. Hernández P. E.. (2013). Antimicrobial activity, antibiotic susceptibility and virulence factors of lactic acid bacteria of aquatic origin intended for use as probiotics in aquaculture. BMC Microbiol. 13, 15. 10.1186/1471-2180-13-15 23347637 PMC3574848

[B54] Nisiotou A. Dourou D. Filippousi M. E. Banilas G. Tassou C. (2014). *Weissella uvarum* sp. nov., isolated from wine grapes. Int. J. Syst. Evol. Microbiol. 64(Pt 11), 3885–3890. 10.1099/ijs.0.066209-0 25180092

[B55] O'Connell J. Schulz-Trieglaff O. Carlson E. Hims M. M. Gormley N. A. Cox A. J. (2015). NxTrim: optimized trimming of Illumina mate pair reads. Bioinformatics 31, 2035–2037. 10.1093/bioinformatics/btv057 25661542

[B56] Oh S. J. Shin N. R. Hyun D. W. Kim P. S. Kim J. Y. Kim M. S.. (2013). *Weissella diestrammenae* sp. nov., isolated from the gut of a camel cricket (*Diestrammena coreana*). Int. J. Syst. Evol. Microbiol. 63(Pt 8), 2951–2956. 10.1099/ijs.0.047548-0 23396715

[B57] Overbeek R. Olson R. Pusch G. D. Olsen G. J. Davis J. J. Disz T.. (2014). The SEED and the Rapid Annotation of microbial genomes using Subsystems Technology (RAST). Nucleic Acids Res. 42, D206–D214. 10.1093/nar/gkt1226 24293654 PMC3965101

[B58] Padonou S. W. Nielsen D. S. Hounhouigan J. D. Thorsen L. Nago M. C. Jakobsen M. (2009). The microbiota of Lafun, an African traditional cassava food product. Int. J. Food Microbiol. 133, 22–30. 10.1016/j.ijfoodmicro.2009.04.019 19493582

[B59] Padonou S. W. Schillinger U. Nielsen D. S. Franz C. M. A. P. Hansen M. Hounhouigan J. D.. (2010). *Weissella beninensis* sp. nov., a motile lactic acid bacterium from submerged cassava fermentations, and emended description of the genus *Weissella*. Int. J. Syst. Evol. Microbiol. 60(Pt 9), 2193–2198. 10.1099/ijs.0.014332-0 19897612

[B60] Prado G. K. S. Torrinha K. C. Cruz R. E. Gonçalves A. B. B. Silva C. A. V. Oliveira F. M. S.. (2020). *Weissella paramesenteroides* WpK4 ameliorate the experimental amoebic colitis by increasing the expression of MUC-2 and the intestinal epithelial regeneration. J. Appl. Microbiol. 129, 1706–1719. 10.1111/jam.14671 32320114

[B61] Praet J. Meeus I. Cnockaert M. Houf K. Smagghe G. Vandamme P. (2015). Novel lactic acid bacteria isolated from the bumble bee gut: *Convivina intestini* gen. *nov., sp. nov., Lactobacillus bombicola sp. nov.*, and *Weissella bombi sp*. nov. Antonie Van Leeuwenhoek. 107, 1337–1349. 10.1007/s10482-015-0429-z 25783976

[B62] Ricciardi A. Parente E. Zotta T. (2009). Modelling the growth of *Weissella cibaria* as a function of fermentation conditions. J. Appl. Microbiol. 107, 1528–1535. 10.1111/j.1365-2672.2009.04335.x 19426261

[B63] Rodriguez-R L. M. Gunturu S. Harvey W. T. Rosselló-Mora R. Tiedje J. M.. (2018). The microbial genomes atlas (MiGA) webserver: taxonomic and gene diversity analysis of archaea and bacteria at the whole genome level. Nucleic Acids Res. 46, W282–W288. 10.1093/nar/gky467 29905870 PMC6031002

[B64] Rodriguez-R L. M. Konstantinidis K. T. (2016). The enveomics collection: a toolbox for specialized analyses of microbial genomes and metagenomes. Peer J. Prepr. 4, e1900ve1901. 10.7287/peerj.preprints.1900v1

[B65] Sandes S. Figueiredo N. Pedroso S. Sant'Anna F. Acurcio L. Abatemarco Junior M.. (2020). *Weissella paramesenteroides* WpK4 plays an immunobiotic role in gut-brain axis, reducing gut permeability, anxiety-like and depressive-like behaviors in murine models of colitis and chronic stress. Food Res. Int. 137, 109741. 10.1016/j.foodres.2020.109741 33233306

[B66] Seemann T. (2014). Prokka: rapid prokaryotic genome annotation. Bioinformatics 30, 2068–2069. 10.1093/bioinformatics/btu153 24642063

[B67] Sica M. G. Olivera N. L. Brugnoni L. I. Marucci P. L. López-Cazorla A. C. Cubitto M. A. (2010). Isolation, identification and antimicrobial activity of lactic acid bacteria from the Bahía Blanca estuary. Rev. Biol. Mar. Oceanogr. 45, 389–397. 10.4067/S0718-19572010000300003

[B68] Snauwaert I. Papalexandratou Z. De Vuyst L. Vandamme P. (2013). Characterization of strains of *Weissella fabalis* sp. nov. and *Fructobacillus tropaeoli* from spontaneous cocoa bean fermentations. Int. J. Syst. Evol. Microbiol. 63(Pt 5), 1709–1716. 10.1099/ijs.0.040311-0 22922535

[B69] Srionnual S. Yanagida F. Lin L. H. Hsiao K. N. Chen Y. S. (2007). Weissellicin 110, a newly discovered bacteriocin from *Weissella cibaria* 110, isolated from plaa-som, a fermented fish product from thailand. Appl. Environ. Microbiol. 73, 2247–2250. 10.1128/AEM.02484-06 17293526 PMC1855655

[B70] Stamatakis A. (2014). RAxML version 8: a tool for phylogenetic analysis and post-analysis of large phylogenies. Bioinformatics 30, 1312–1313. 10.1093/bioinformatics/btu033 24451623 PMC3998144

[B71] Tatusova T. DiCuccio M. Badretdin A. Chetvernin V. Nawrocki E. P. Zaslavsky L.. (2016). NCBI prokaryotic genome annotation pipeline. Nucleic Acids Res. 44, 6614–6624. 10.1093/nar/gkw569 27342282 PMC5001611

[B72] Teixeira C. G. Fusieger A. Milião G. L. Martins E. Drider D. Nero L. A.. (2021). *Weissella*: an emerging bacterium with promising health benefits. Probiotics Antimicrob. Proteins 13, 915–925. 10.1007/s12602-021-09751-1 33565028

[B73] Tofalo R. Fusco V. Böhnlein C. Kabisch J. Logrieco A. F. Habermann D.. (2020). The life and times of yeasts in traditional food fermentations. Crit. Rev. Food Sci. Nutr. 60, 3103–3132. 10.1080/10408398.2019.1677553 31656083

[B74] Tomasik P. Horton D. (2012). Enzymatic conversions of starch. Adv. Carbohydr. Chem. Biochem. 68, 59–436. 10.1016/B978-0-12-396523-3.00001-4 23218124

[B75] van der Maarel M. J. van der Veen B. Uitdehaag J. C. Leemhuis H. Dijkhuizen L. (2002). Properties and applications of starch-converting enzymes of the alpha-amylase family. J. Biotechnol. 94, 137–155. 10.1016/S0168-1656(01)00407-2 11796168

[B76] Wang C. Zhang C. W. Chen H. C. Yu Q. Pei X. F. Liu H. C. (2008). [Phylogeny analysis and identification of two bacterial strains sourcing from human intestine and having resistance to acid and bile]. Sichuan Da Xue Xue Bao Yi Xue Ban. 39, 263–366. Available online at: https://europepmc.org/article/med/18630699 18630699

[B77] Wang J. P. Yoo J. S. Jang H. D. Lee J. H. Cho J. H. Kim I. H. (2011). Effect of dietary fermented garlic by *Weissella koreensis* powder on growth performance, blood characteristics, and immune response of growing pigs challenged with *Escherichia coli* lipopolysaccharide. J. Anim. Sci. 889, 2123–2131. 10.2527/jas.2010-3186 21317348

[B78] Wang W. Liu W. Chu W. (2020). Isolation and preliminary screening of potentially probiotic *Weissella confusa* strains from healthy human feces by culturomics. Microb. Pathog. 147, 104356. 10.1016/j.micpath.2020.104356 32610159

[B79] Wang Y. Wu J. Lv M. Shao Z. Hungwe M. Wang J.. (2021). Metabolism characteristics of lactic acid bacteria and the expanding applications in food industry. Front. Bioeng. Biotechnol. 9, 612285. 10.3389/fbioe.2021.612285 34055755 PMC8149962

[B80] Wangpaiboon K. Laohawuttichai P. Kim S. Y. Mori T. Nakapong S. Pichyangkura R.. (2021). A GH13 α-glucosidase from *Weissella cibaria* uncommonly acts on short-chain maltooligosaccharides. Acta Crystallogr. D. Struct. Biol. 77(Pt 8), 1064–1076. 10.1107/S205979832100677X 34342279

[B81] Wolter A. Hager A. S. Zannini E. Czerny M. Arendt E. K. (2014). Influence of dextran-producing Weissella cibaria on baking properties and sensory profile of gluten-free and wheat breads. Int. J. Food Microbiol. 172, 83–91. 10.1016/j.ijfoodmicro.2013.11.015 24361837

[B82] Xiong L. Ni X. Niu L. Zhou Y. Wang Q. Khalique A.. (2019). Isolation and preliminary screening of a *Weissella confusa* strain from Giant Panda (*Ailuropoda melanoleuca*). Probiotics Antimicrob. Proteins. 11, 535–544. 10.1007/s12602-018-9402-2 29654473

[B83] Yeu J. E. Lee H. G. Park G. Y. Lee J. Kang M. S. (2021). Antimicrobial and antibiofilmactivities of *Weissella cibaria* against pathogens of upper respiratory tract infections. Microorganisms 9, 1181. 10.3390/microorganisms9061181 34070813 PMC8229644

[B84] Yin Y. Mao X. Yang J. Chen X. Mao F. Xu Y. (2012). dbCAN: a web resource for automated carbohydrate-active enzyme annotation. Nucleic Acids Res. 40, W445–W451. 10.1093/nar/gks479 22645317 PMC3394287

[B85] Yu H. S. Lee N. K. Choi A. J. Choe J. S. Bae C. H. Paik H. D. (2019). Anti-inflammatory potential of probiotic strain *Weissella cibaria* JW15 Isolated from Kimchi through regulation of NF-κB and MAPKs pathways in LPS-induced RAW 264.7 cells. J. Microbiol. Biotechnol. 29, 1022–1032. 10.4014/jmb.1903.03014 31216608

[B86] Zannini E. Jeske S. Lynch K. M. Arendt E. K. (2018). Development of novel quinoa-based yoghurt fermented with dextran producer *Weissella cibaria* MG1. Int. J. Food Microbiol. 268, 19–26. 10.1016/j.ijfoodmicro.2018.01.001 29316448

[B87] Zannini E. Mauch A. Galle S. Gänzle M. Coffey A. Arendt E. K.. (2013). Barley malt wortfermentation by exopolysaccharide-forming *Weissella cibaria* MG1for the production of a novel beverage. J. Appl. Microbiol. 115, 1379–1387. 10.1111/jam.12329 23957391

[B88] Zartl B. Silberbauer K. Loeppert R. Viernstein H. Praznik W. Mueller M. (2018). Fermentation of non-digestible raffinose family oligosaccharides and galactomannans by probiotics. Food Funct. 9, 1638–1646. 10.1039/C7FO01887H 29465736

