# Supplementary material for: Novel Insights Into the Phylogeny and Biotechnological Potential of Weissella Species
Source: Front Microbiol. 2022 Jun 22;13:914036. doi: 10.3389/fmicb.2022.914036 (PMC9257631; doi:10.3389/fmicb.2022.914036)
Supplement: Supplementary Table S1 — List of Weissella strains used in this study. [file Table_1.docx]

| **Supplementary Table S1.** List of *Weissella* strains used in this study | | | | |
| --- | --- | --- | --- | --- |
| *species* | **type strain** | **Reference Accession** | **16S rRNA** | **Assigned by** |
| *W. beninensis* | DSM 22752^T^ | JAGMVS010000000 | EU439435 | Padonou et al. 2010 |
| *W. bombi* | DSM 28794^T^ | NZ_FMAO00000000.1 | LK054487 | Praet et al., 2015 |
| *W. ceti* | CCUG 59653^T^ | NZ_ANCA01000000.1 | FN813251 | Vela et al., 2011 |
| *W. cibaria* | CCUG 41967^T^ | NZ_VNGZ01000000.1 | AJ295989 | Bjorkroth et al., 2002 |
| *W. coleopterum* | HDW19^T^ | NZ_CP049888.1 | MN099422 | Hyun et al., 2021 |
| *W. confusa* | ATCC 10881^T^ | NZ_CP027563.1 | AB023241 | Collins et al., 1993 |
| *W. cryptocerci* | 26KH-42^T^ | NZ_CP037940.1 | MK395366 | Heo et al., 2019 |
| *W. diestrammenae* | DSM 27940^T^ | JAGMVT010000000 | JQ646523 | Oh et al., 2013 |
| *W. fabalis* | LMG 26217^T^ | JAGMVU010000000 | HE576795 | Snauwaert et al., 2013 |
| *W. fabaria* | LMG 24286^T^ | JAGMVV010000000 | FM179678 | De Bruyne et al., 2010 |
| *W. ghanensis* | DSM 19935^T^ | JAGMVW010000000 | AM882997 | De Bruyne et al., 2008 |
| *W. halotolerans* | ATCC 35410^T^ | NZ_ATUU01000000.1 | AB022926 | Collins et al.,1993 |
| *W. hellenica* | ATCC 51523^T^ | NZ_JAAXPM010000000.1 | X95981 | Collins et al.,1993 |
| *W. jogaejeotgali* | FOL01^T^ | NZ_CP014332.1 | KP027016 | Lee et al., 2015 |
| *W. kandleri* | ATCC 51149^T^ | NZ_JQBP01000000.1 | AB022922 | Collins et al.,1993 |
| *W. koreensis* | CCUG 47134^T^ | NZ_AKGG01000000.1 | AY035891 | Lee et al., 2002 |
| *W. minor* | ATCC 35412^T^ | NZ_JQCD01000000.1 | AB022920 | Collins et al.,1993 |
| *W. muntiaci* | 8 H-2^T^ | NZ_SDGZ01000000.1 | MK774696 | Lin et al., 2020 |
| *W. oryzae* | DSM 25784^T^ | NZ_DF820484.1 | AB690345 | Tohno et al., 2013 |
| *W. paramesenteroides* | ATCC 33313^T^ | ACKU01000000.1 | X95982 | Collins et al.,1993 |
| *W. sagaensis* | CCM 8924^T^ | BLKA01000001.1 | LC438526 | Li et al., 2020 |
| *W. soli* | CCUG 46608^T^ | NZ_CP017326.1 | AY028260 | Magnusson et al., 2002 |
| *W. thailandensis* | CCUG 46557^T^ | NZ_BJEC01000000.1 | MT760016 | Tanasupawat et al., 2000 |
| *W. uvarum* | B18NM42^T^ | JAGMVX010000000 | KF999666 | Nisiotou et al., 2014 |
| *W. viridescens* | ATCC 12706^T^ | NZ_CP061835.1 | X52568 | Collins et al.,1993 |

**References**

Björkroth, K.J., Schillinger, U., Geisen, R., Weiss, N., Hoste, B., Holzapfel, W.H., Korkeala, H.J., Vandamme, P. (2002). Taxonomic study of *Weissella confusa* and description of *Weissella cibaria* sp. nov., detected in food and clinical samples. Int. J. Syst. Evol. Microbiol. 52(Pt 1), 141-148. doi: 10.1099/00207713-52-1-141.

Collins, M.D., Samelis, J., Metaxopoulos, J., Wallbanks, S. (1993). Taxonomic studies on some Leuconostoc-like organisms from fermented sausages: description of a new genus *Weissella* for the *Leuconostoc paramesenteroides* group of species. J. Appl. Bacteriol. 75, 595-603. doi: 10.1111/j.1365-2672.1993.tb01600.x.

De Bruyne, K., Camu, N., De Vuyst, L., Vandamme, P. (2010). *Weissella fabaria* sp. nov., from a Ghanaian cocoa fermentation. Int. J. Syst. Evol. Microbiol. 60, 1999-–2005. doi: 10.1099/ijs.0.019323-0.

De Bruyne, K., Camu, N., Lefebvre, K., De Vuyst, L., Vandamme, P. (2008). *Weissella ghanensis* sp. nov., isolated from a Ghanaian cocoa fermentation. . Int. J. Syst. Evol. Microbiol. 58, 2721–2725. . doi: 10.1099/ijs.0.65853-0.

Heo, J., Hamada, M., Cho, H., Weon, H.Y., Kim, J.S., Hong, S.B., Kim, S.J., Kwon, S.W. (2019). *Weissella cryptocerci* sp. nov., isolated from gut of the insect *Cryptocercus kyebangensis*. Int. J. Syst. Evol. Microbiol. 69(9), 2801-2806. doi: 10.1099/ijsem.0.003564.

Hyun, D.W., Lee, J.Y., Sung, H., Kim, P.S., Jeong, Y.S., Lee, J.Y., Yun, J.H., Choi, J.W., Han, J.E., Lee, S.Y., et al. (2021). *Brevilactibacter coleopterorum* sp. nov., isolated from the intestine of the dark diving beetle Hydrophilus acuminatus, and *Weissella coleopterorum* sp. nov., isolated from the intestine of the diving beetle *Cybister lewisianus*. Int. J. Syst. Evol. Microbiol. 71, 4779 doi: 10.1099/ijsem.0.004779.

Lee, S.H., Ku, H.J., Ahn, M.J., Hong, J.S., Lee, S.H., Shin, H., Lee, K.C., Lee, J.S., Ryu, S., Jeon, C.O., et al. (2015). *Weissella jogaejeotgali* sp. nov., isolated from jogaejeotgal, a traditional Korean fermented seafood. Int. J. Syst. Evol. Microbiol. 65, 4674-4681. doi: 10.1099/ijsem.0.000631.

Lee JS, Lee KC, Ahn JS, Mheen TI, Pyun YR, Park YH. *Weissella koreensis* sp. nov., isolated from kimchi. *Int J Syst Evol Microbiol* 2002; **52**:1257-1261

Li, Y.Q., Tian, W.L., Gu, C.T. (2020). *Weissella sagaensis* sp. nov., isolated from traditional Chinese yogurt. Int. J. Syst. Evol. Microbiol. 70, 2485-2492. doi: 10.1099/ijsem.0.004062.

Lin, S.T., Wang, L.T., Wu, Y.C., Guu, J.J., Tamura, T., Mori, K., Huang, L., Watanabe, K. (2020). *Weissella muntiaci* sp. nov., isolated from faeces of Formosan barking deer (*Muntiacus reevesi*). Int. J. Syst. Evol. Microbiol. 70(3), 1578-1584. doi: 10.1099/ijsem.0.003937.

Oh, S.J., Shin, N.R., Hyun, D.W., Kim, P.S., Kim, J.Y., Kim, M.S., Yun, J.H., Bae, J.W. (2013). *Weissella diestrammenae* sp. nov., isolated from the gut of a camel cricket (*Diestrammena coreana*). Int. J. Syst. Evol. Microbiol. 63(Pt 8), 2951-2956. doi: 10.1099/ijs.0.047548-0.

Magnusson, J., Jonsson, H., Schnürer, J., Roos, S. (2002). *Weissella soli* sp. nov., a lactic acid bacterium isolated from soil. Int. J. Syst. Evol. Microbiol. 52(Pt 3), 831-834. doi: 10.1099/00207713-52-3-831.

Nisiotou, A., Dourou, D., Filippousi, M.E., Banilas, G., Tassou, C. (2014). *Weissella uvarum* sp. nov., isolated from wine grapes. Int. J. Syst. Evol. Microbiol. 64(Pt 11), 3885-3890. doi: 10.1099/ijs.0.066209-0.

Padonou, S.W., Schillinger, U., Nielsen, D.S., Franz, C.M.A.P., Hansen, M., Hounhouigan, J.D., Nago, M.C., Jakobsen, M. (2010). *Weissella beninensis* sp. nov., a motile lactic acid bacterium from submerged cassava fermentations, and emended description of the genus *Weissella*. Int. J. Syst. Evol. Microbiol. 60(Pt 9), 2193-2198. doi: 10.1099/ijs.0.014332-0.

Praet, J., Meeus, I., Cnockaert, M., Houf, K., Smagghe, G., Vandamme, P. (2015). Novel lactic acid bacteria isolated from the bumble bee gut: *Convivina intestini* gen. nov., sp. nov., *Lactobacillus bombicola* sp. nov., and *Weissella bombi* sp. nov. Antonie Van Leeuwenhoek. 107, 1337-1349. doi: 10.1007/s10482-015-0429-z.

Snauwaert, I., Papalexandratou, Z., De Vuyst, L., Vandamme, P. (2013). Characterization of strains of *Weissella fabalis* sp. nov. and *Fructobacillus tropaeoli* from spontaneous cocoa bean fermentations. Int. J. Syst. Evol. Microbiol. 63(Pt 5), 1709-1716. doi: 10.1099/ijs.0.040311-0.

Tanasupawat, S., Shida, O., Okada, S., Komagata, K. (2000). *Lactobacillus acidipiscis* sp. nov. and *Weissella thailandensis* sp. nov., isolated from fermented fish in Thailand. Int. J. Syst. Evol. Microbiol. 50(Pt 4), 1479-1485. doi: 10.1099/00207713-50-4-1479.

Tohno, M., Kitahara, M., Inoue, H., Uegaki, R., Irisawa, T., Ohkuma, M., Tajima, K. (2013). *Weissella oryzae* sp. nov., isolated from fermented rice grains. Int. J. Syst. Evol. Microbiol. 63(Pt 4), 1417-1420. doi: 10.1099/ijs.0.043612-0.

Vela, A.I., Fernández, A., Bernaldo de Quirós, Y., Herráez, P., Domínguez, L., Fernández-Garayzábal, J.F. (2011). *Weissella ceti* sp. nov., isolated from beaked whales (Mesoplodon bidens). Int. J. Syst. Evol. Microbiol. 61(Pt 11), 2758-2762. doi: 10.1099/ijs.0.028522-0.
