# Supplementary figures and images for: Novel Insights Into the Phylogeny and Biotechnological Potential of Weissella Species
Source: Front Microbiol. 2022 Jun 22;13:914036. doi: 10.3389/fmicb.2022.914036 (PMC9257631; doi:10.3389/fmicb.2022.914036)

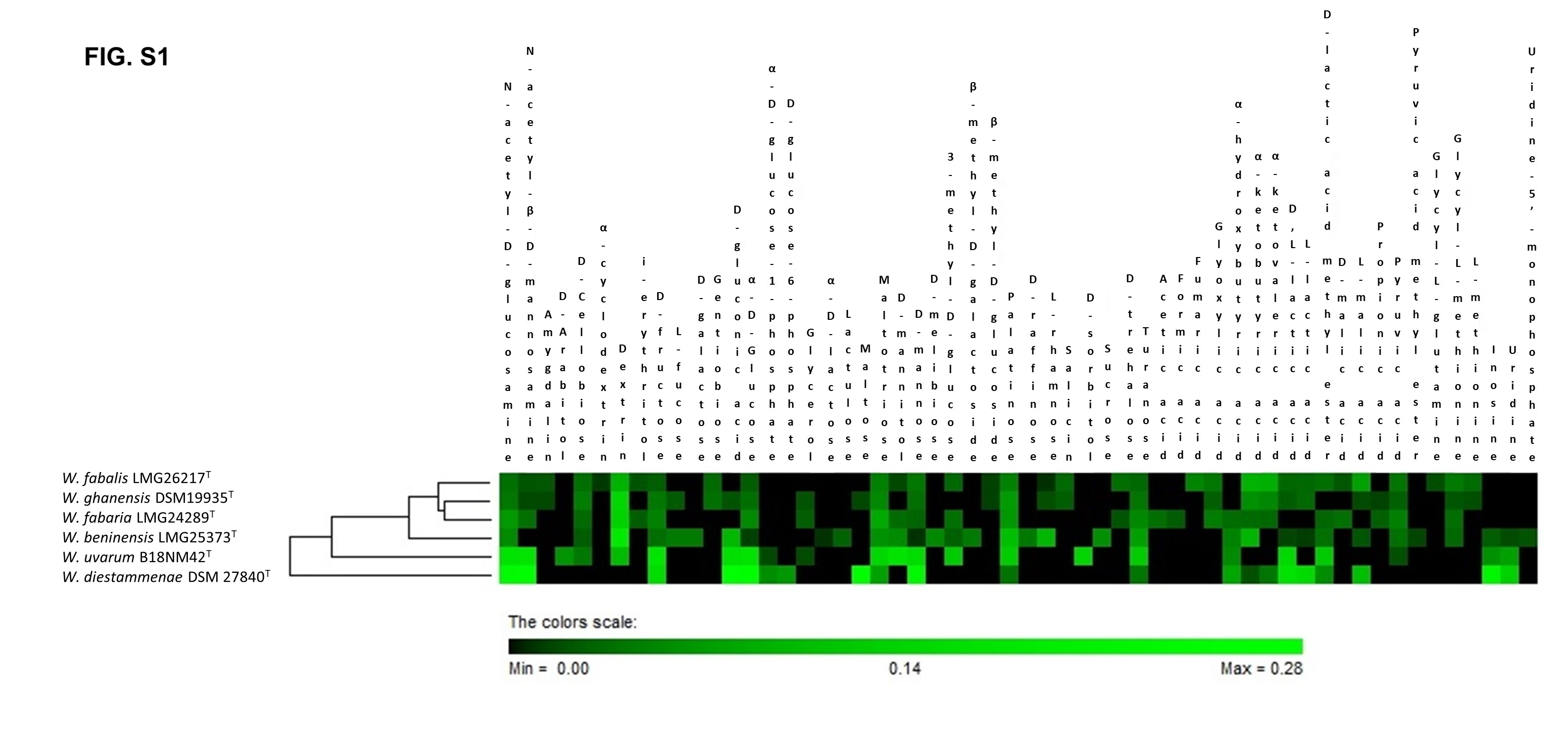

Supplement: Supplementary Figure S1 — Cluster analysis of Weissella strains based on carbon source consumption by PermutMatrix. [file Image_1.tif]
